# *Spartan*: A Comprehensive Tool for Understanding Uncertainty in Simulations of Biological Systems

**DOI:** 10.1371/journal.pcbi.1002916

**Published:** 2013-02-28

**Authors:** Kieran Alden, Mark Read, Jon Timmis, Paul S. Andrews, Henrique Veiga-Fernandes, Mark Coles

**Affiliations:** 1 Centre for Systems Biology, School of Biosciences, University of Birmingham, Birmingham, United Kingdom; 2 Department of Electronics, University of York, York, United Kingdom; 3 Department of Computer Science, University of York, York, United Kingdom; 4 Instituto de Medicina Molecular, Faculdade de Medicina de Lisboa, Lisboa, Portugal; 5 Centre for Immunology and Infection, University of York and Hull York Medical School, York, United Kingdom; University of Wisconsin-Madison, United States of America

## Abstract

Integrating computer simulation with conventional wet-lab research has proven to have much potential in furthering the understanding of biological systems. Success requires the relationship between simulation and the real-world system to be established: substantial aspects of the biological system are typically unknown, and the abstract nature of simulation can complicate interpretation of *in silico* results in terms of the biology. Here we present *spartan* (Simulation Parameter Analysis R Toolkit ApplicatioN), a package of statistical techniques specifically designed to help researchers understand this relationship and provide novel biological insight. The tools comprising *spartan* help identify which simulation results can be attributed to the dynamics of the modelled biological system, rather than artefacts of biological uncertainty or parametrisation, or simulation stochasticity. Statistical analyses reveal the influence that pathways and components have on simulation behaviour, offering valuable biological insight into aspects of the system under study. We demonstrate the power of *spartan* in providing critical insight into aspects of lymphoid tissue development in the small intestine through simulation. *Spartan* is released under a GPLv2 license, implemented within the open source R statistical environment, and freely available from both the Comprehensive R Archive Network (CRAN) and http://www.cs.york.ac.uk/spartan. The techniques within the package can be applied to traditional ordinary or partial differential equation simulations as well as agent-based implementations. Manuals, comprehensive tutorials, and example simulation data upon which *spartan* can be applied are available from the website.

This is a *PLOS Computational Biology* Software Article

## Introduction

The integration of computer simulation with current experimental techniques has become a popular approach to aid the understanding of biological systems [1]. Computational techniques permit exploration of the underlying biological data on which a simulation is based, and complement wet-lab research by facilitating in silico experimentation impractical or impossible to perform using current methods [2]–[4]. Simulations are however abstractions of the biological systems they capture, and this separation must be appreciated in the interpretation of in silico results. Such simulation results may be affected by uncertainty arising from aspects of the biological system that are currently unknown and need to be assumed, and by uncertainty introduced in the implementation of the simulator [5]. We recently noted that for a majority of simulation results in the biological literature, little attempt is made to elucidate how representative a simulation result is in terms of the biological system captured [6]. Uncertainty and sensitivity analyses have, however, found recent application in exploring the behaviour of biological simulations to appreciate the effect of uncertainty on simulation results [6]–[10].

Whereas a number of packages have been developed that aid simulation development [11], [12] to the best of our knowledge there is no comprehensive package available for determining how representative a simulation is of its biological system and understanding how in silico results can be interpreted in the context of the biological domain. As such, we have developed spartan (Simulation Parameter Analysis R Toolkit ApplicatioN), a toolkit of statistical techniques that aid understanding and analysis of results generated through simulation. *Spartan* is freely available, open-source, and implemented within the R statistical environment. The package provides implementations of previously described statistical analysis techniques [6], [7], [13] that when brought together provide a comprehensive toolkit to explore the effect uncertainty has on simulation results. Such uncertainty may be present in two forms: aleatory uncertainty that arises through stochasticity inherent in both the biological and simulated systems, and epistemic uncertainty reflecting simulation parameters for which a value has yet to be or cannot be determined as the biological understanding is incomplete [5]. Appreciating this effect is critical for interpreting simulation results with respect to the biological system under study [6]. The use of statistical tools provides evidence of which simulation results can be ascribed to the dynamics of the model of the biological system implemented, an important consideration for retaining confidence in the results of in silico experimentation.

In previous work we have utilised computer simulation to model the process of lymphoid tissue development [9], [10]. Here we demonstrate the use of s*partan* in an exploration of results generated from this simulator: to determine the number of simulation samples required to mitigate stochastic effects and attain a desired level of experimental accuracy, build confidence that our results are representative of biology as opposed to parameterisation artefacts resulting from epistemic uncertainty, and gain valuable biological insight through rigorous statistical analysis of simulation results. Whereas our previous work highlights the need to adhere to a robust method of simulation design and development informed by wet-lab research, our case study shows how spartan can provide a mechanism to integrate simulation results back into wet-lab research.

## Design and Implementation

S*partan* was created to provide a comprehensive toolkit of previously described statistical techniques. Four techniques are included, each providing a different means of analysing simulation data to understand the effect of uncertainty on results and to provide biological insight. The first two techniques have been previously described [6], but this package includes the first available implementation of these techniques. *Spartan* assumes that calibration has been performed to establish the parameter values that produce a baseline simulation behaviour [6]. Such behaviour is measured through a set of simulation outputs (responses) that are of biological interest and can be qualitatively compared with the biological system. Uncertainty and sensitivity analyses can then determine the effects of parameter perturbations on simulation responses. As each technique utilises different parameter sampling methods, it is not possible to use the results generated for one technique for any other technique currently in the package. Although Technique 1 is specifically designed for use with stochastic simulations (such as agent-based implementations), techniques 2–4 can be applied for both mathematical (ODE/PDE) and agent-based implementations. For techniques 2–4, spartan provides methods to generate parameter values and analyse the output generated from them. For Technique 4 (eFAST), *spartan* includes a bespoke implementation of the eFAST algorithm rather than makes use of available methods in other packages for reasons of consistency and ability to analyse a range of output from different simulation implementations. As it is not assumed that simulation data is normally distributed, all statistical comparisons that establish significance between sets of simulation responses are performed using non-parametric tests.

### 1. Consistency (or Aleatory) Analysis: Understanding Effect of Aleatory Uncertainty

Prior to any simulator being used as a tool to complement wet-lab investigations, it is critical that the effect of inherent simulation stochasticity on results be understood [5]. To illustrate, in agent-based simulations the use of pseudo-random number generators in dictating agent behaviour can produce different simulation results despite use of identical parameter values. To mitigate the effects of this uncertainty and achieve representative in silico results, replicate simulation runs are necessary. A technique developed by Read et al. [6] is provided that establishes the number of replicate runs required to achieve a desired level of experimental accuracy. In contrast to the other techniques in spartan, this need only be applied to stochastic simulation systems.

Consistency analysis operates by contrasting distributions of simulation responses, all generated using the same fixed set of parameter values and containing identical numbers of simulation samples. By varying the number of samples comprising the distributions, the analysis determines the number required to obtain statistically consistent distributions. Larger sample sizes produce increasingly identical distributions, thereby mitigating the effect of simulation stochasticity on results. In the description by Read et al [6], 20 distributions are generated and contrasted for each sample size, but this can be varied within *spartan* to suit particular applications.

As an example, one could consider analysing sample sizes of 5, 50, 100, and 300 to determine the number of simulation runs required to mitigate aleatory uncertainty. A set of parameter values is fixed and used for all runs. The researcher performing the analysis must then gather 20 sets of simulation results for each sample size being analysed, each containing that number of results. In this example, where a sample size of 5 is being examined, 20 sets of simulation results should be generated, with each containing 5 sets of simulation results. Where a sample size of 300 is being analysed, each of the 20 sets should contain the results of 300 runs. When this is complete, each sample size is analysed in turn. A distribution of median responses for each simulation run is generated for each of the 20 subsets.

Distributions 2–20 are contrasted with the distribution from the 1st set using the Vargha-Delaney A-Test [14], a non-parametric effect magnitude test that establishes scientific significance by contrasting two populations of samples and returning the probability that a randomly selected sample from one population will be larger than a randomly selected sample from the other. Statistical significance is determined by comparing this result with measures set by the authors of the test: results above 0.71 or below 0.29 indicate a scientifically significant difference between the populations, and 0.5 indicates no difference [14]. However *spartan* provides the option to change these magnitude effects if required. A suitable sample size is found where there is no statistical difference between the 1st set of results and the remaining 19 sets. This statistical difference can be seen in plots *spartan* produces for each sample size. A further plot is produced detailing the maximum A-Test score observed for each sample size, easing identification of the number of runs required to minimise difference between simulation results and thus mitigate aleatory uncertainty.

### 2. Robustness Analysis: Determining a Simulation's Robustness to Parameter Perturbation

Any biological simulation will feature biologically-derived parameters for which values are fully or partially unknown: some biological values cannot be determined experimentally, whereas others cannot be represented easily within a simulation. For example, diffusion of a chemoattractant could be implemented using a particular mathematical function for which values cannot be verified, as biological quantities cannot currently be measured. Robustness analysis examines the implications of biological uncertainty or parameter estimation on simulation results. Where a simulation is found to be highly sensitive to such parameters caution must be exercised when interpreting results; they may be artefacts of parametrisation rather than representations of the biology [5].

Robustness to parameter perturbation is explored using a ‘one at a time’ approach [6]: each parameter is adjusted independently of others, which retain their calibrated values. The Vargha-Delaney A-Test described previously [14] is employed to determine if changing a parameter value leads to scientifically significant behavioural alteration in contrast to the baseline simulation. This indicates how robust the simulator is to alteration of each parameter, and the points at which parameter perturbations result in significant changes in simulation behaviour. Confidence in the validity of simulation results can be gauged by contrasting this information with biologically accepted ranges of values. The A-Test results for each parameter are presented on a plot, providing easy identification of parameter values that cause a scientifically significant change in simulation response.

### 3. Global Sensitivity Analysis: Identification of Compound Effects through Simultaneously Perturbing All Parameters

Though robustness analysis elucidates affects of perturbing single parameters, it cannot reveal compound effects that occur when two or more are adjusted simultaneously. The effect one parameter has may rely on the value of another. Global sensitivity analyses reveal such effects, showing how different parameters could be coupled, and can indicate the parameters that have the greatest influence on simulation responses. *Spartan* includes a sampling-based technique that perturbs the values of all parameters of interest simultaneously [6], [7], [13] based on a provided range of values for each parameter. Through simultaneous perturbation of parameters, the results of this analysis are highly representative of simulation dynamics. Highly influential parameters indicate the pathways and components that have a substantial effect on simulation behaviour, and in identifying such relationships, this analysis has the potential to offer unique biological insight into the system the model describes. This has the potential to inform future wet-lab investigations by suggesting specific biological components to focus upon.

A latin-hypercube sampling approach is used to select parameter sets from within these ranges, whilst minimising correlations in parameter values across the sets and ensuring an efficient coverage of parameter space [13]. The methodology used in sampling is described in detail by Read et al [6]. Simulations are executed for each parameter set generated, and simulation response values determined. Where necessary these responses will represent an averaged response over a number of runs for a particular set of parameter values. A plot is produced for each parameter-response pairing, revealing correlations between parameter and response values, which are quantified through calculation of Partial Rank Correlation Coefficients (PRCC) and are reported on the plot header. PRCCs account for non-linear relationships between parameter and response, and correct for the effects of other parameters on the response, giving a robust indication of the effect this parameter has on simulation response although others are also being perturbed.

### 4. eFAST: Partitioning Output Variance between Input Parameters

The extended Fourier Amplitude Sampling Test (eFAST) developed by Saltelli et al [15], [16] also a global analysis technique, is a variance decomposition method that partitions the simulation output variance caused by an alteration in parameter values between the input parameters. This provides a statistical measure revealing the proportion of variance that can be explained by perturbing the value of each factor, and thus determines how sensitive the simulation and biological system is to that parameter. Through applying this technique, highly influential pathways can be identified as potential targets for future laboratory investigation.

For each simulation parameter included in this analysis, a range over which values are to be explored is provided. Taking each in turn, values are chosen for all parameters through the use of sinusoidal functions of a particular frequency through the parameter space, with the frequency of the parameter of interest being varied greatly to that used for its complementary set. A number of parameter values are selected from points along each of these curves, creating a set of simulation parameters for each parameter of interest. An illustration of this sampling approach can be seen in Marino *et al* [7]. The authors note that due to the symmetrical properties of sinusoidal functions, it is possible that the same parameter value sets could be selected. To address this, a re-sampling scheme is encouraged where a phase shift is introduced into each frequency, and sampling repeated using a slightly different curve [7]. Selection of the number of re-sample curves and parameter values chosen from the curves is an important aspect of running this algorithm, and it is suggested that the user makes themselves familiar with equations provided in Marino et al [7] that aid this decision. Consequently, a number of parameter value sets are created for each parameter of interest, for a particular curve. This process is repeated for an extra parameter, the ‘dummy’, which has an arbitrary value range but no impact on simulation behaviour. This enables a comparison between the impact of each parameter and one known to have no effect on simulation response. As an example, for 7 parameters, plus a dummy, three re-sample curves, and 65 parameter values from points along the curves, 1,560 sets of parameters would be produced. Spartan produces a csv file for each parameter of interest and each curve, containing the parameter value sets on which simulations should be executed. Thus in this example, twenty-four files would be produced, each containing sixty five sets of parameters. For analyses where a large number of parameters are explored, this technique could be computationally expensive [17], [18].

Simulation results are analysed taking into account the frequencies that were used to generate the parameter set used. Through Fourier analysis using these frequencies, variation in output can be partitioned between the parameters, giving an indication of the impact each has on simulation response. Using the equations given in Marino et al [7], two sensitivity indexes are calculated for each parameter-response pairing: a first-order (Si) and total order sensitivity (STi) index. The first indicates the fraction of output variance in that response that can be explained by the value assigned to the parameter. The latter indicates the variance in that response caused by higher-order non-linear effects between the parameter and the others under investigation. Although the dummy parameter has no influence on simulation dynamics, the algorithm may assign this parameter non-zero sensitivity indexes due to aliasing or interference effects, or simplifying assumptions the technique makes in calculating each STi index [7]. Reasoning for this is given in Supplemental material that accompanies the description of the technique in Marino et al [7]. A determination of whether a parameter has a significant impact on simulation response is made by examining the sensitivity indexes, contrasting these with the indexes calculate for the ‘Dummy’ parameter. As this is contrasting sensitivity indexes rather than simulation responses, a statistical measure is generated using a two-sample t-test. Spartan produces both a csv file with these statistics and a plot for each simulation response, detailing the Si and STi indexes for each parameter.

## Results

### The Case Study

The example simulation data, from which the results presented here were generated, is taken from our recently published lymphoid tissue development simulator (http://www.cs.york.ac.uk/immunesims/frontiers) [9], [10]. An agent-based simulation was developed that captures an abstraction of the early stages of the biological process (12 hours into a 72 hour period). The simulation responses are cell velocity and displacement measures taken at various time-points. All output data and tutorials that reproduce each result described are available at www.cs.york.ac.uk/spartan.

In our published analyses [9], [10] and examples presented here, we focus on six parameters for which a value remains unknown, each constrained to the following respective range: chemoThreshold (0–1), chemoLowerLinearAdjust (0.015–0.08), chemoUpperLinearAdjust (0.1–0.5), thresholdBindProbability (0–1), vcamSlope (0.25–2), and maxVCAMeffectProbabilityCutoff (0–1).

### 1. Consistency (or Aleatory) Analysis: Understanding Effect of Aleatory Uncertainty

**Figure 1:**
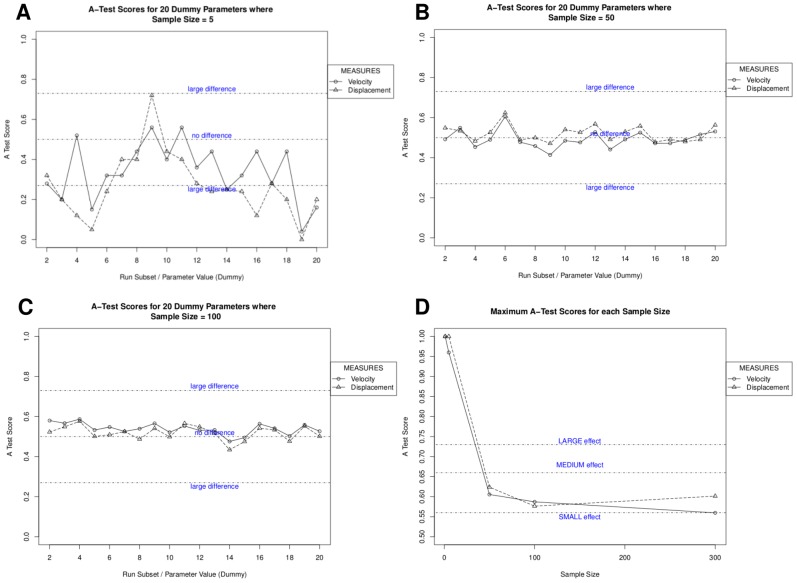
Use of *spartan* to mitigate aleatory uncertainty in stochastic simulations. *Spartan's* consistency analysis technique that can determine the number of runs required to generate a representative result from a stochastic simulation. A, B, C: A-Test scores for sample sizes of 5, 50, and 300 runs respectively. D. Maximum A Test score for each simulation response over 20 result sets for all sample sizes analysed. Scores below 0.5 are assigned corresponding values above 0.5 as direction of effect is not important. The effect magnitude thresholds are indicated.

To determine the number of simulation runs required to obtain a representative result, we analysed sample sizes of 1, 5, 50, 100, 300, and 500 runs. Parameter values were kept constant at their calibrated values. Each sample size is analysed in turn using the procedure described, with the generation of 20 subsets of each sample size. This analysis thus required 19,120 individual runs. The online tutorial examines the first five sample sizes. *Spartan* produces the plots in Figure 1, where Figures 1(a,b,c) show the A-Test scores for all simulation output responses in each of the 20 result sets, for 5, 50, and 100 samples respectively. Figure 1(d) shows the maximum A Test score for each simulation response over the 20 result sets, for all sample sizes analysed. The latter indicates that reducing the effect magnitude of aleatory uncertainty on simulation results to less than ‘small’ (the desired level) requires more than 300 samples when compiling results, thus a sample size of 500 was chosen. It is important to note however that this is specific to our simulation, and unlikely to apply in all cases where *spartan* is applied. A full analysis for this simulation is found in Patel et al [9]; the online tutorial and results in Figure 1 stop at 300 runs to ensure the tutorial data is of manageable size for download.

### 2. Robustness Analysis: Determining a Simulation's Robustness to Parameter Perturbation

In our case study [9], we analysed the six simulation parameters for which biological values are currently unknown using the robustness analysis. The online tutorial demonstrates how both the parameter samples and results described in this section have been generated for two of these parameters.

#### Parameter sampling

Parameter value sets for the six parameters were created using the methods in the *spartan* package. These are output as comma separated value files, one for each parameter, then post-processed into a format that can be read by the simulator. The sampling method begins at the parameters lower value and increases the value by a set increment until the upper limit is reached. For the six parameters under examination here, the increments used were: chemoThreshold (0.1), chemoLowerLinearAdjust (0.005), chemoUpperLinearAdjust (0.05), thresholdBindProbability (0.1), vcamSlope (0.25), and maxVCAMeffectProbabilityCutoff (0.1).

#### Analysis

Each parameter is addressed in turn, and simulation results for each value assigned to that parameter analysed. 500 simulation executions are performed for each parameter value in accordance with consistency analysis results. In our case, this resulted in 32,500 individual simulation runs. The distribution of response values obtained for each parameter value is contrasted with a distribution obtained using baseline parameter values using the Vargha-Delaney A-Test [14].

**Figure 2:**
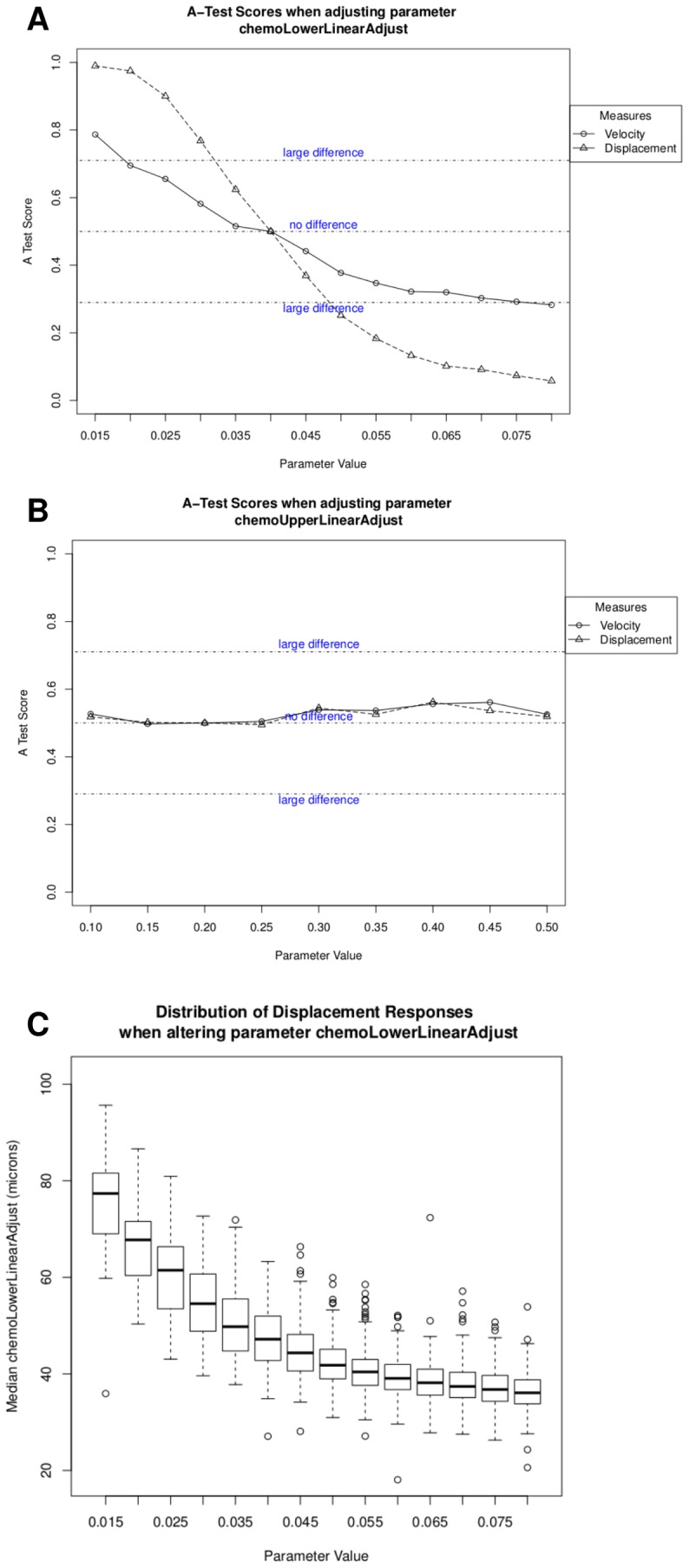
Use of *spartan* to determine the simulators robustness to parameter perturbation. A: A-Test scores for simulations perturbing the initial expression level of a chemoattractant. This parameter has a large effect on both simulation responses. B: A-Test scores for simulations perturbing the upper limit of chemoattractant expression, which when perturbed has no significant effect on simulation response. C: Distribution of cell displacement responses for the parameter perturbed in A.

*Spartan* produced the plots in Figure 2, where Figures 2(a,b) show the A-Test scores for an alteration in the values of two simulation parameters that model expression of chemoattractant molecules. The x-axis details the range of values explored and the y-axis shows the A test scores obtained by contrasting response values for perturbed parameter values with calibrated values. Figure 2(c) shows the effect that adjusting the value of the parameter in 2(a) has on cell displacement as a box-plot of response distributions. Results suggest that a change in the initial expression of chemoattractant molecules has a statistically significant effect on simulation response, and is more critical than the upper limit of expression, which has no statistically significant impact. This suggests that the initial expression level of chemoattractant molecules is an important factor influencing cell behaviour at this time-point in development. Laboratory investigations could then examine this experimentally through blocking chemokine expression at certain time-points in development, to determine if this prediction holds.

### 3. Global Sensitivity Analysis: Identification of Compound Effects through Simultaneously Perturbing All Parameters

In this analysis we sought to identify any compound effects that become apparent when the values of the six parameters examined in Technique 2 above are perturbed simultaneously. This has revealed the parameters that are highly influential on simulation behaviour, and provided unique biological insight into the factors that are important at this stage of tissue development. The online tutorial demonstrates how both the parameter samples and results described in this section have been generated.

#### Parameter sampling

Using the latin-hypercube sampling approach, 500 sets of simulation parameter values were generated.

#### Analysis

Five hundred parameter value sets were generated from the parameter space using the latin-hypercube sampling approach. With results from Technique 1 suggesting 500 simulation executions are required to gain a representative result from our simulation, a total of 250,000 simulation executions were performed to generate the data required for this analysis. Median output responses for each of the parameter value sets were then calculated from the 500 sets of results. Taking each parameter in turn, median response values are plotted against the parameter value that generated them, and partial rank correlation coefficients are calculated.

**Figure 3:**
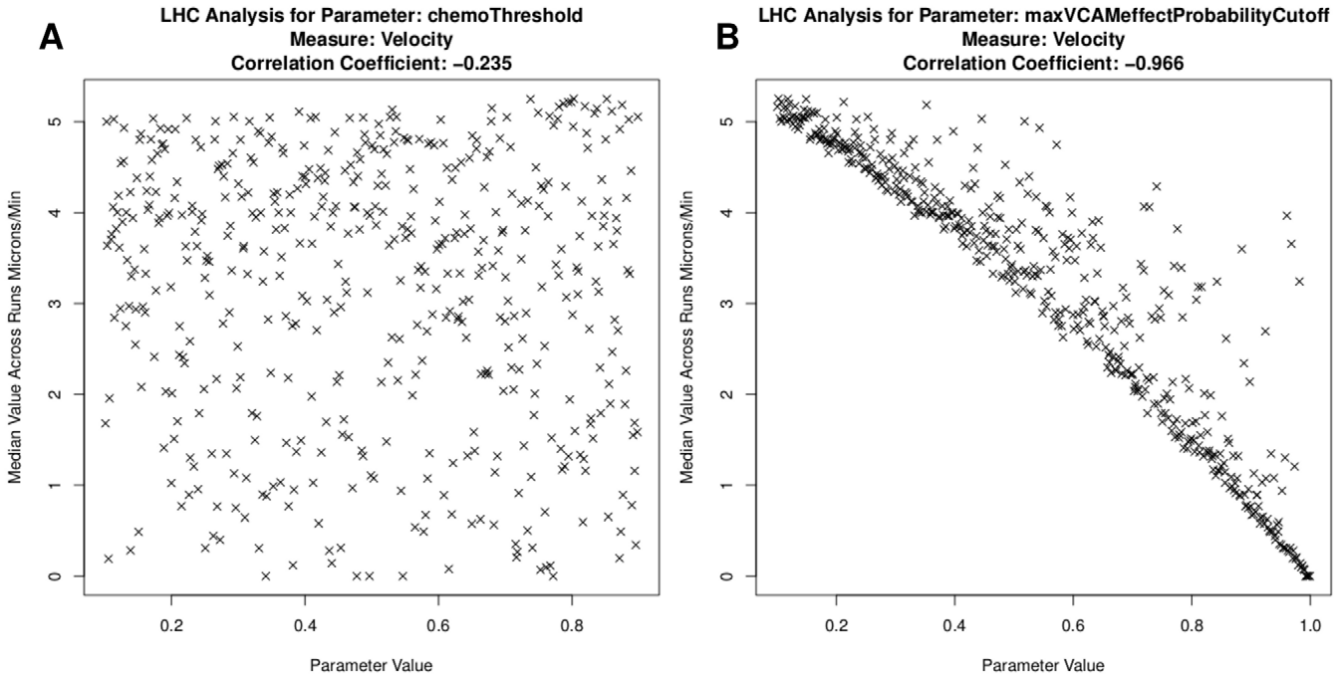
Use of *spartan* to identify compound effects between parameters. A: Parameter that captures the chemoattractant expression level required to influence cell motility. No trend or effects are apparent. B: Parameter which captures the level of adhesion required to restrict cell motility. A clear trend is apparent suggesting this has a large influence on simulated cell behaviour.

For online tutorial 3, *spartan* produces the plots in Figure 3. These detail cell velocity responses for two parameters. In Figure 3(b), detailing the effect of cellular adhesion, a clear trend emerges. The correlation coefficient reveals this parameter's significant influence on the simulation response. The same conclusions cannot be drawn for the parameter in Figure 3(a), where no trend emerges. Whereas the previous technique identified initial chemokine expression as an influential factor when the parameters where perturbed individually, this result suggests adhesion factor expression is the highly influential pathway at this time-point. Such a prediction could be verified experimentally through examining cell behaviour when expression of adhesion factors are blocked.

### 4. eFAST: Partitioning Output Variance between Input Parameters

In this analysis we examined the same six parameters as above, and determined the proportion of variation in simulation response that can be explained by perturbing the value of each parameter. Through use of the eFAST approach [7], [16] we have determined how sensitive the simulation is to each parameter, and thus suggested the impact of each biological factor on tissue development. The online tutorial demonstrates how both the parameter samples and results described in this section have been generated.

#### Sampling

Parameter value sets have been generated using the sinusoidal curve sampling approach. We have seven parameters (six plus the ‘dummy’ used for statistical comparison), taken 65 parameter values from each curve, and employed three re-sampling curves, producing 1,365 parameter value sets, 195 per parameter.

#### Analysis

**Figure 4:**
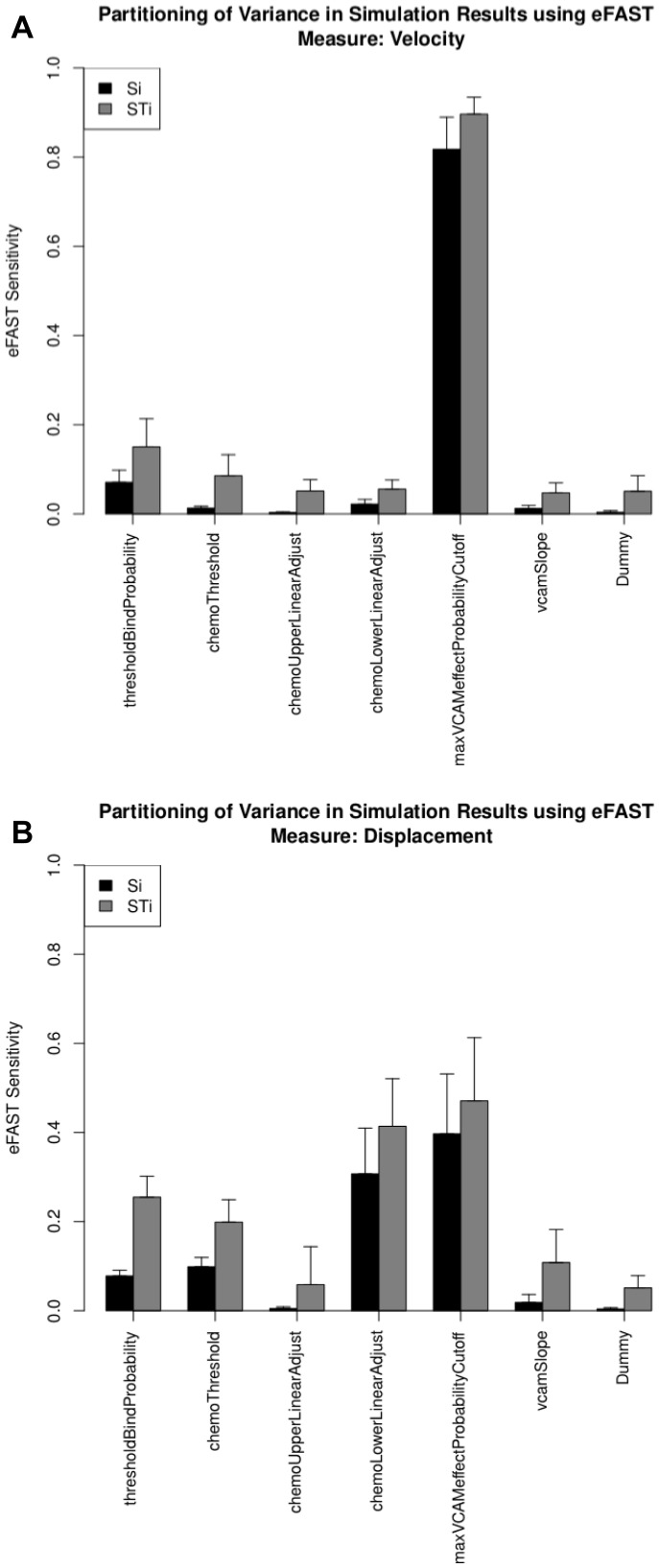
Use of eFAST method within *spartan* to partition variance in simulation results between parameters. Si (black): the fraction of output variance that can be explained by the value assigned to that parameter; STi (grey): the variance caused by higher-order non-linear effects between that parameter and the others explored. Error bars are standard error over three resample curves. A: Velocity response. B: Displacement response.

Simulation responses are analysed using the Fourier frequency approach [7], [15]. 500 runs were performed for each set of parameter values, as suggested by results generated using Technique 1, and median responses calculated. With 1,365 individual parameter sets, this analysis therefore required 682,500 runs in our case. Plots are created for each simulation output response (velocity and displacement), detailing the first-order (Si) and total-order (STi) sensitivity indexes calculated for each parameter of interest. Indications of significance of each parameters sensitivity index, contrasted to those calculated for the ‘Dummy’ parameter using a two-sample t-test, are output to a CSV file in the directory specified by the user prior to running the analysis. For online tutorial 4, spartan produces the plots in Figure 4. In our published study [9], we utilised our simulator and statistical methods described in techniques one to three, and determined no significant role for chemoattractant factors at an early stage of tissue development. Results shown in Figure 4 examine the same time-point with this additional analysis method, and support these conclusions. We suggest that the factor that influences cell velocity at this early stage in development is the level of expression of cellular adhesion factors (maxVCAMeffectProbabilityCutoff parameter). This supports predictions made by use of Technique 3, but counters the accepted view in the literature, where chemokines are known to have an influential role in tissue development [19], [20]. However results in the literature draw these conclusions from an examination of the whole tissue development time-period (72 hours), rather than just the early stage (12 hours) focused on here and in our previous study [9]. Thus potentially our analysis, using spartan, suggests that different factors could be important at different stages of development. Examining a later time-point in development, both in the lab and through use of *spartan*, may suggest that this is indeed the case, and the tissue development period is more complex than previously thought.

## Availability and Future Directions

*Spartan* has been developed and runs within the platform-independent R statistical environment (version 2.13.1 or greater), and can be freely downloaded from http://www.cs.york.ac.uk/spartan or from the Comprehensive R Archive Network (CRAN). The package is open source and available under the GNU General Public License (GPLv2). The release of *spartan* as an R package rather than as standalone software allows simulation developers to build *spartan* into their analysis routine and extend the analysis with methods provided in additional packages where necessary. Manuals, comprehensive tutorials, and simulation data used are available from the website. *Spartan* remains in on-going development in parallel to further simulation case studies being developed in our group, and thus further suitable sensitivity analysis techniques will be added when appropriate. In similarity to recent advances in simulation development toolkits [11], [12], a graphical user interface will also shortly be released for *spartan* and made available on the website, aiding use of the tool for use unfamiliar with R.

## Supporting Information

Software S1 Spartan R package for Linux and Mac OS. Includes tutorials for each technique.
(ZIP)

Software S2 Spartan R package for Windows OS. Includes tutorials for each technique.
(ZIP)

## References

[1] Germain RN, Meier-schellersheim M, Nita-lazar A, Fraser IDC (2011) Systems Biology in Immunology: A Computational Modeling Perspective. Annual Review of Immunology 29: 527–585. 10.1146/annurev-immunol-030409-101317 PMC3164774 21219182

[2] Efroni S, Harel D, Cohen IR (2003) Toward Rigorous Comprehension of Biological Complexity: Toward Rigorous Comprehension of Biological Complexity: Modeling, Execution, and Visualization of Thymic T-Cell Maturation. Genome Research 13: 2485–2497. 14597657 10.1101/gr.1215303 PMC403768

[3] Kirschner DE, Linderman JJ (2009) Mathematical and computational approaches can complement experimental studies of host-pathogen interactions. Cellular Microbiology 11: 531–539. 19134115 10.1111/j.1462-5822.2008.01281.x PMC2720090

[4] Andrews PS, Polack FAC, Sampson AT, Stepney S, Timmis J (2010) The CoSMoS Process, Version 0.1: A Process for the Modelling and Simulation of Complex Systems. Technical Report YCS-2010-453. Department of Computer Science, University of York 1–40.

[5] Helton JC (2008) Uncertainty and sensitivity analysis for models of complex systems. In: Barth TJ, Griebel M, Keyes DE, Nieminen RM, Roose D, et al.., editors. Computational Methods in Transport: Verification and Validation. Springer. pp. 207–228.

[6] Read M, Andrews PS, Timmis J, Kumar V (2012) Techniques for Grounding Agent-Based Simulations in the Real Domain: a case study in Experimental Autoimmune Encephalomyelitis. Mathematical and Computer Modelling of Dynamical Systems 18: 67–86.

[7] Marino S, Hogue IB, Ray CJ, Kirschner DE (2008) A methodology for performing global uncertainty and sensitivity analysis in systems biology. Journal of theoretical biology 254: 178–196. 18572196 10.1016/j.jtbi.2008.04.011 PMC2570191

[8] Ray JCJ, Flynn JL, Kirschner DE (2009) Synergy between individual TNF-dependent functions determines granuloma performance for controlling mycobacterium tuberculosis infection. Journal of theoretical biology 182: 3706–3717. 10.4049/jimmunol.0802297 PMC3182770 19265149

[9] Patel A, Harker N, Moreira-Santos L, Ferreira M, Alden K, et al (2012) Differential RET responses orchestrate lymphoid and nervous enteric system development. Science Signalling 5: ra55. 10.1126/scisignal.2002734 22855506

[10] Alden K, Timmis J, Andrews PS, Veiga-Fernandes H, Coles MC (2012) Pairing experimentation and computational modelling to understand the role of tissue inducer cells in the development of lymphoid organs. Frontiers in Immunology 3: 172. 22826707 10.3389/fimmu.2012.00172 PMC3399454

[11] Puzone R, Kohler B, Seiden P, Celada F (2002) IMMSIM, a flexible model for in machina experiments on immune system responses. Future Generation Computer Systems 18: 961–972.

[12] Meier-Schellersheim M, Xu X, Angermann B, Kunkel EJ, Jin T, et al (2006) Key role of local regulation in chemosensing revealed by a new molecular interaction-based modeling method. PLoS Computational Biology 2: 710–724. 10.1371/journal.pcbi.0020082 PMC1513273 16854213

[13] Saltelli A, Chan K, Scott EM (2000) Sensitivity Analysis. Wiley series in probability and statistics. Wiley

[14] Vargha A, Delaney HD (2000) A critique and improvement of the CL Common Language Effect Size Statistics of McGraw and Wong. Journal of Educational and Behavioural Statistics 25: 101–132.

[15] Saltelli A, Bollardo R (1998) An alternative way to compute Fourier amplitude sensitivity test (FAST). Comput Stat Data Anal 26: 445–460.

[16] Saltelli A (2004) Sensitivity Analysis in practice: A guide to assessing scientific models. Wiley

[17] Tarantola S, Gatelli D, Mara TA (2006) Random balance designs for the estimation of first order global sensitivity indices. Reliability Engineering & System Safety 91: 717–727.

[18] Ratto M, Pagano A, Young P (2007) State Dependent Parameter metamodelling and sensitivity analysis. Computer Physics Communications 177: 863–876.

[19] Randall TD, Carragher DM, Rangel-Moreno J (2008) Development of secondary lymphoid organs. Annual Review Immunology 26: 627–650. 10.1146/annurev.immunol.26.021607.090257 PMC2590644 18370924

[20] Luther SA, Ansel KM, Cyster JG (2003) Overlapping roles of CXCL13, interleukin 7 receptor alpha, and CCR7 ligands in lymph node development. The Journal of experimental medicine 197: 1191–1198. 12732660 10.1084/jem.20021294 PMC2193976

