# Correction: *Spartan*: A Comprehensive Tool for Understanding Uncertainty in Simulations of Biological Systems

**DOI:** 10.1371/annotation/cfcaffb6-8515-4595-b6cc-85b84703d7d5

**Published:** 2013-08-12

**Authors:** Kieran Alden, Mark Read, Jon Timmis, Paul S. Andrews, Henrique Veiga-Fernandes, Mark Coles

1) Two of the corresponding author's three affiliations were missing. Kieran Alden's full affiliations are as follows:

- Centre for Systems Biology, School of Biosciences, University of Birmingham, Birmingham, United Kingdom

- Department of Computer Science, University of York, York, United Kingdom

- Centre for Immunology and Infection, University of York and Hull York Medical School, York, United Kingdom

2) The 3rd and 6th authors should be listed as co-corresponding authors. Jon Timmis can be contacted at jon.timmis@york.ac.uk and Mark Coles at mark.coles@york.ac.uk. 

