# Supplementary material for: Spartan: A Comprehensive Tool for Understanding Uncertainty in Simulations of Biological Systems
Source: PLoS Comput Biol. 2013 Feb 28;9(2):e1002916. doi: 10.1371/journal.pcbi.1002916 (PMC3585389; doi:10.1371/journal.pcbi.1002916)
Supplement: Software S1 — Spartan R package for Linux and Mac OS. Includes tutorials for each technique. (ZIP) [file pcbi.1002916.s001.zip › Technique4_Tutorial.pdf]

Tutorial on using the *spartan* package to analyse agent-based simulation results

Technique 4: Parameter Sampling and Analysis through use of the extended Fourier Amplitude Sampling Test (eFAST)

1 Introduction

spartan, or (**S**imulation **P**arameter **A**nalysis **R** **T**oolkit **A**pplicatio**N**) is an R package which aids the understanding of the effect aleatory and epistemic uncertainty have on the output from a simulation. This set of tutorials makes use of available example simulation output to demonstrate how a variety of methods can be applied to further understand the results that have been generated. Following through each example should make it easier to apply the tookit to results generated by any agent-based computer simulation. This tutorial focuses on the partitioning of variance in simulation results between input parameters

2 The *spartan* Package

Computer simulations are becoming a popular technique to use in attempts to further our understanding of complex systems. This package provides code for four techniques described in available literature which aid the analysis of simulation results, at both single and multiple timepoints in the simulation run. The first technique addresses aleatory uncertainty in the system caused through inherent stochasticity, and determines the number of replicate runs necessary to generate a representative result. The second examines how robust a simulation is to parameter perturbation, through the use of a one-at-a-time parameter analysis technique. Thirdly, a latin hypercube based sensitivity analysis technique is included which can elucidate non-linear effects between parameters and indicate implications of epistemic uncertainty with reference to the system being modelled. Finally, a further sensitivity analysis technique, the extended Fourier Amplitude Sampling Test (eFAST) has been included to partition the variance in simulation results between input parameters, to determine the parameters which have a significant effect on simulation behaviour.

3 The Case Study

The example simulation results have been taken from an ongoing project which seeks to understand the formation of lymphoid tissue in the small intestine. This simulation outputs cell behaviour measures at various points in the simulation and measures describing the development of the tissue, which occurs through interactions between the cells. Techniques 2-4 of this package allow us to explore how input parameter value affects the behaviour of these cells. We need Technique 1 to tell us how many simulation runs we need for each condition explored to ensure we have a robust representative result.

4 Scope

Do note that the idea of this tutorial is to demonstrate the application of the toolkit, and is not intended to act as a full introduction to using Sensitivity Analysis techniques in the analysis of simulation results. Where useful, links to further reading have been included.

5 Prerequisites

- The R statistical environment, version 2.13.1 or later.
- The spartan R package, downloaded from the Comprehensive R Archive Network (CRAN) or from the project website.
- The lhs and gplots R packages, available for download from CRAN.
- The example simulation data for this tutorial, available from the project website.
- From version 1.2 of *spartan*, simulation results can be in either CSV or XML format. For earlier versions, results must be pre-processed to be in CSV format.

6 Running Technique 4: eFAST Sampling and Analysis

This technique analyses simulation results generated through parametering using the eFAST approach (extended Fourier Amplitude Sampling Test, Saltelli et al, reference below). This perturbs the value of all parameters at the same time, with the aim of partitioning the variance in simulation output between input parameters. Values for each parameter are chosen using fourier frequency curves through a parameters potential range of values. A selected number of values are selected from points along the curve. Though all parameters are perturbed simultaneously, the method does focus on one parameter of interest in turn, by giving this a very different sampling frequency to that assigned to the other parameters. Thus for each parameter of interest in turn, a sampling frequency is assigned to each parameter and values chosen at points along the curve. So a set of simulation parameters then exists for each parameter of interest. As this is the case, this method can be computationally expensive, especially if a large number of samples is taken on the parameter search curve, or there are a large number of parameters. On top of this, to ensure adequate sampling each curve is also resampled with a small adjustment to the frequency, creating more parameter sets on which the simulation should be run. This attempts to limit any correlations and limit the effect of repeated parameter value sets being chosen. Thus, for a system where 8 parameters are being analysed, and 3 different sample curves used, 24 different sets of parameter value sets will be produced. Each of these 24 sets then contains the parameter values chosen from the frequency curves. This number of samples should be no lower than 65 (see the Marino paper for an explanation of how to select sample size). The explanation of this is greatly aided by an example, which is covered later in this tutorial.

Once the sampling has been performed, a number of simulation runs should be performed for each set generated (this number that which has become apparent through analysis of aleatory uncertainty, or use of Technique 1 within the *spartan* package). The eFAST algorithm then examines the simulation results for each parameter value set and, taking into account the sampling frequency used to produce those parameter values, partitions the variance in output between the input parameters.

The *spartan* package includes methods to both create parameter value samples using fourier frequency sampling, and to analyse the simulation results. This tutorial covers both methods.

7 Parameter Sampling

The package contains a method which can produce a parameter space sample for the parameters of interest. Simulations should then be run on each of the generated parameter sets. This is done as follows:

1. Open a text editor (gedit or similar). Now we are going to declare the R variables required by the package to produce the parameter value sets. Type or copy in the text below. Firstly, the *spartan* library is imported. The variables required for this analysis are then declared in capital letters. The line underneath each, beginning with a #, is a description of that being declared. Make sure you set the FILEPATH parameter correctly to match the folder where you would like the parameter value sets to be output to.

```
library(spartan)
# Import the package

FILEPATH<-"/media/FreeAgent/package_Test_Data/eFAST/Sampling"
# The folder where the results should be output to
NUMCURVES <- 3
# Number of resample curves to use in the sampling procedure
PARAMETERS <- c("thresholdBindProbability","chemoThreshold",
"chemoUpperLinearAdjust","chemoLowerLinearAdjust",
"maxVCAMeffectProbabilityCutoff","vcamSlope","Dummy")
# Names of the parameters to generate parameter value samples for.
# Should be within an array
NUMSAMPLES <- 65
# The number of parameter sample sets to create for each curve
PMIN<-c(0,0.10,0.10,0.015,0.1,0.25,1)
# The minimum value in the range for each parameter
PMAX<-c(100,0.9,0.50,0.08,1.0,5.0,10)
# The maximum value in the range for each parameter
```

2. To get the value sets for each parameter, the following method is used. Copy the text below into the R script file below the declarations you have made above:

```
efast_generate_sample(FILEPATH,NUMCURVES,NUMSAMPLES,PARAMETERS,
PMIN,PMAX)
```

3. Save the file with a suitable filename and .R extension (for example eFAST_Sampling.R)

4. For Linux or Mac OS, open a command prompt, and navigate to the directory where this file was saved. Type the following:

```
Rscript eFAST_Sampling.R
```

If you are using Windows, open R and type the following into the R Command Prompt (where [*path to directory* is the full path to where eFAST_Sampling.R is saved]:

```
source("C:\[\textit{path to directory}]\eFAST_Sampling.R")
```

This will produce a large number of CSV files. Remember eFAST works by examining one parameter of interest at a time, going through the sample space using a significantly different frequency to that at which the others are sampled. Thus, for each resample curve, one set of parameter value sets is produced for each parameter of interest. Thus, in this case, we have 3 resample curves and 7 parameters (including the 'dummy'), so 24 different sets of 65 parameter samples are produced. This explains why the analysis is so computationally expensive, as this is 1560 different parameter sets on which simulations will need to be performed. Then, for each set, a number of runs are performed to produce a robust

result which considers the effect of Aleatory Uncertainty (see Technique 1). In our case, we know we need 300 runs per set of parameters to produce a robust result, meaning a total of 468,000 simulation runs.

Each CSV file is named with the convention Curve[*Sample_Curve_Number*]_[*Parameter_Name*].csv

8 Parameter Analysis

This section of the tutorial performs this analysis for the lymphoid tissue formation simulation. In this case, we are going to examine two cell behaviour measures, Velocity and Displacement, that are captured for a period representing one hour of real time, to determine how a change in parameter value influences this behaviour. In this tutorial, only a brief overview of the technique is provided. For a full explanation of how this analysis is performed, we suggest reading the Marino and Saltelli references below.

The easiest way to explain this is through the use of an example. In our case, we have six simulation parameters of interest. As the eFAST analysis makes use of a 'Dummy' parameter when determining which are significant, this therefore becomes seven. For each of the parameters in turn, the sampling algorithm above has generated 65 parameter value sets using a fourier frequency approach. The algorithm then performed the sampling another two times for each parameter, which slightly adjusted the frequency used, to ensure more adequate sampling. Thus we have 3 sampling curves, 7 parameters, and for each curve, 65 parameter value sets chosen. Thus a total of 1,560 different parameter value sets. As stated in the sampling section above, repeat simulation runs were then performed for each, meaning a total of 468,000 sets of data to analyse. We now look at how the toolkit analyses this data:

1. Download the eFAST example data from the project website and extract the results.
2. The first thing to note is the folder structure. To use this method, the simulation results do need to be in a specific format (Figure 1 eFAST folder structure). The structure has four levels:
   (i) Folders for each of the curves being explored. Thus, for our example, we have 3 curves, so three folders, numbered 1-3.
   (ii) Folders for each of the parameters of interest, for that curve. In the example case, there are seven parameters, so 7 folders, numbered 1-7.
   (iii) Folders for each set of parameter values being analysed, for that parameter of interest using this sampling curve. In the example case, the parameter space was sampled 65 times, thus there are 65 folders, numbered 1-65.
   (iv) A folder for each of the simulation results where the simulation was run under those conditions. Will match the number of runs required that was determined through Aleatory Analysis (Technique 1). So, in our case this was 300, so there are 300 folders numbered 1-300.
3. With this data available, open a text editor (gedit or similar). Now we are going to declare the R variables required to run this analysis. Type or copy in the text on Page 6. Firstly, the *spartan* library is imported. The variables required for this analysis are then declared in capital letters. The line underneath each beginning with a #, is a description of that being declared. Make sure you set the FILEPATH parameter correctly to state where the simulation results have been extracted to.

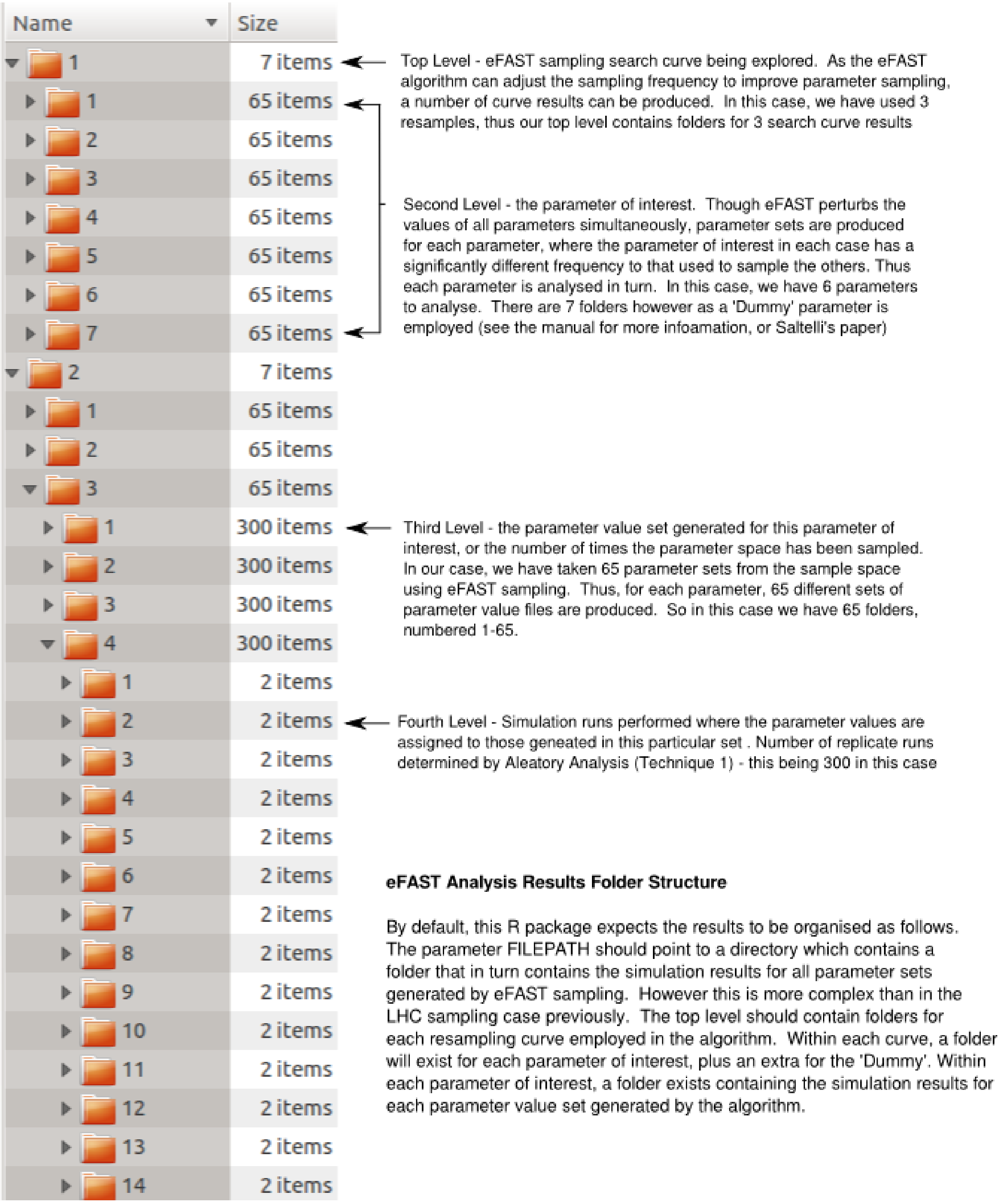

Figure 1: Simulation results folder structure that should exist for use with this tool

```
library(spartan)
# Import the package
library(gplots)
# Import the graphing package

FILEPATH<-"/media/FreeAgent/package_Test_Data/eFAST/Results"
# Folder containing the simulation results
NUMCURVES<-3
# Number of resample curves employed when the parameter space was
# sampled
PARAMETERS <- c("thresholdBindProbability","chemoThreshold",
"chemoUpperLinearAdjust","chemoLowerLinearAdjust",
"maxVCAMeffectProbabilityCutoff","vcamSlope","Dummy")
# Array of the parameters to be analysed
NUMSAMPLES<-65
# The number of parameter value sets created in latin-hypercube
# sampling
NUMRUNSPERSAMPLE<-300
# Number of runs performed for each parameter value set
MEASURES<-c("Velocity","Displacement")
# The simulation output measures being examined
RESULTFILEFORMAT<-"csv"
# Simulation result file format. Should be either CSV or XML
RESULTFILENAME<-"trackedCells_Close"
# The output file containing the simulation results from that simulation run
# Note no file extension
ALTERNATIVEFILENAME<-NULL
# Not used in this case, but this is useful in cases where two result files may
# exist (for example if tracking cells close to an area, and those further away
#  two output files could be used).  Here, results in a second file are processed
# if the first is blank or does not exist.
OUTPUTCOLSTART<-10
# Used with CSV result file formats
# The column within the csv results file where the results start.  This is useful
# as it restricts what is read in to R, getting round potential errors where the
# first column contains an agent label (as R does not read in CSV files where the
# first column contains duplicates)
OUTPUTCOLEND<-11
# Used with CSV result file formats
# Last column of the output measure results
MEDIANSFILEFORMAT<-"csv"
# File format of median results file that will be generated in this tutorial.
# Should be either XML or CSV. This is used as, for some applications, a simulation
# results set for processing may have been generated using methods other than
# spartan, and may not be in CSV format.
MEDIANSFILENAME<-"EgSet_Medians"
# For each parameter value set being analysed, a file is created containing the
# median of each output measure, of each simulation run for that value. This sets
# the name of this file. Note no file extension
CURVERESULTSFILENAME<-"EgSet_AllResults"
# Name of the summary file for each eFAST curve, showing the value assigned to
# each parameter and the simulation outcome median(s) when run under that condition
```

```
# Note no file extension
EFASTRESULTFILENAME<-"EgSet_eFAST_Analysis"
# Name of the final result file for this analysis, showing the partitioning of
# the variance between input parameters
# Note no file extension
OUTPUTMEASURES_TO_TTEST<-1:2
# Which of the output measures to T-Test for significance (if not all)
TTEST_CONF_INT<-0.95
# T-Test confidence level
GRAPH_FLAG<-TRUE
# Boolean to note whether summary graphs should be produced
TIMEPOINTS<-NULL; TIMEPOINTSCALE<-NULL
# Not used in this case, but when a simulation is analysed at multiple timepoints
# (see later in tutorial)
```

4. Now to examine the first of the four methods (we are going to do each individually in the tutorial so the functionality becomes apparent but in reality you will run all three methods one after another in the same text file). Copy the below into the text file under the above declarations:

```
efast_generate_medians_for_all_parameter_subsets(FILEPATH,
NUMCURVES,PARAMETERS,NUMSAMPLES,NUMRUNSPERSAMPLE,MEASURES,
RESULTFILEFORMAT,RESULTFILENAME,ALTERNATIVEFILENAME,
OUTPUTCOLSTART,OUTPUTCOLEND,MEDIANSFILEFORMAT,
MEDIANSFILENAME)
```

As stated previously and in other tutorials for this package, a robust representative result is achieved when the simulation is run a number of times for the same parameter conditions. The aim of this method is to examine each parameter value set and produce a file containing the median of each output measure for each simulation run performed under those conditions. In our example case, we know we achieve a representative result if we perform 300 runs of the simulation under the same criteria. The aim therefore is to produce a file which contains the medians of each of our simulation output measures, of each of the 300 runs. We can then use this distribution in comparison with that produced by another parameter value set.
In this case, we examine each resampling curve in turn, and each parameter in turn. Each parameter then has 65 sets of value sets generated in sampling, with each containing 300 simulation runs. When this method is invoked, we will produce a set of medians for each of these 65 value sets, for each parameter. These will be stored in the third level of the folder structure, under the filename specified in the R parameter MEDIANSFILENAME, with file format specified by MEDIANSFILEFORMAT

In normal cases, you would now save the text file and run the script in R to generate these files. However, unlike the tutorials on Technique 1 and 2, actual simulation results have not been included in the download due to the excessive file such from such a large number of simulations runs. Instead, the data included is the result of the above method being run. Have a look at a few of the files to ensure you understand the format, but do not run this method else you will get an error in this case. Instead, change the text file so these lines are commented out, like this:

```
#efast_generate_medians_for_all_parameter_subsets(FILEPATH,
# NUMCURVES,PARAMETERS,NUMSAMPLES,NUMRUNSPERSAMPLE,MEASURES,
```

```
# RESULTFILEFORMAT,RESULTFILENAME,ALTERNATIVEFILENAME,
# OUTPUTCOLSTART,OUTPUTCOLEND,MEDIANSFILEFORMAT,
# MEDIANSFILENAME)
```

5. We are now going to run the second method, which takes the set of medians contained in each of the parameter value set folders and produces a file summarising the simulation results. In the same file in the text editor, add the following method call, then save the file.

   ```
   efast_get_overall_medians(FILEPATH,NUMCURVES,PARAMETERS,
   NUMSAMPLES,MEASURES,MEDIANSFILEFORMAT,MEDIANSFILENAME,
   CURVERESULTSFILENAME)
   ```

   This method produces a summary of the results for a particular resampling curve. This shows, for each parameter of interest, the median of each simulation output measure for each of the 65 parameter value sets generated.

   Here's an example. We examine resampling curve 1, and firstly examine parameter 1 (thresholdBindProbability in this case). For this parameter of interest, 65 different parameter value sets were generated from the frequency curves, thus we have 65 different sets of simulation results. The previous method produced a summary showing the median of each output measure for each run. Now, this method calculates the median of these medians, for each output measure, and stores these in the summary. Thus, for each parameter of interest, the medians of each of the 65 sets of results are stored. The next parameter is then examined, until all have been analysed. This produces a snapshot showing the median simulation output for all parameter value sets generated for the first resample curve. These are stored within the second level of the folder structure, with filename as specified by the R variable CURVERESULTSFILENAME.

6. For Linux or Mac OS, open a command prompt, and navigate to the directory where this file was saved. Type the following:

   ```
   Rscript eFAST_Analysis.R
   ```

   If you are using Windows, open R and type the following into the R Command Prompt (where [*path to directory* is the full path to where eFAST_Analysis.R is saved]:

   ```
   source("C:\[\textit{path to directory}]\eFAST_Analysis.R")
   ```

   Navigate through the folder structure and open one of the summary files. You will see there are 65 rows of data, one for each of the parameter sample sets. You will then note there are 14 different columns, two for each parameter of interest (as there are two output measures). Make yourself familiar with the format of the file produced. This will help when applying the toolkit to other simulations.

7. Now that these summary files have been produced, we can use the eFAST analysis algorithm in our attempts to partition the output variance between input parameters. In the same file in the text editor, add the following method call, then save the file.

   ```
   efast_run_Analysis(FILEPATH,MEASURES,PARAMETERS,NUMCURVES,
   NUMSAMPLES,OUTPUTMEASURES_TO_TTEST,TTEST_CONF_INT,
   GRAPH_FLAG,CURVERESULTSFILENAME,EFASTRESULTFILENAME,
   TIMEPOINTS,TIMEPOINTSCALE)
   ```

8. Return to the command prompt and run the R script using the same command as previously.

   As noted previously, it is a good idea to familiarise yourself with the Marino and Saltelli references as these go into greater detail on how this analysis is performed. Rather than repeat that here, we examine the output that the package has produced, and how this can be interpreted.

   Navigate to the top level of the folder structure. You will see that a file EgSet_eFAST_Analysis.csv has been produced. Open this file in a spreadsheet so its structure can be examined. You will notice that the first column contains the parameters that have been explored. For each, there are then a number of statistics, for each output measure. The important values are:

   (i) The Si measure. Si is the first-order sensitivity index (where i is the parameter). Calculated as the simulation variance for that parameter over the unique sampling frequency it was assigned, it represents the fraction of model output variance that is explained by an input variation of a given parameter.
   (ii) The STi measure. STi is the total-order sensitivity index (again i is the parameter). This statistic again indicates the fraction of model output variance, but this time includes any higher-order, non-linear effects between the parameter of interest and its complementary parameters.
   (iii) The SCi measure. SCi is the variance caused by the parameter of interests complementary set (or, all other parameters bar that of interest).
   (iv) Error values. As the analysis has been done for a number of sample curves, it is possible to calculate other statistical measures such as the standard error. This is useful for plotting the results graphically.

   For ease of representation, the method also produces a graph showing this data for each measure. These will have been saved in the top level directory, and should look like those seen in Figure 2 and 3.

   Have a look at the graphs. You will see that the parameters of interest, and the dummy parameter, are on the x axis. A parameters Si and STi value are deemed significant when a comparison is drawn with the Si and STi of the dummy parameter. In other statistical tests a comparison is made with zero, but that is not possible for eFAST (see Marino paper). So instead the dummy is used. This has no influence on simulation results, but will still be assigned an Si and STi value by the algorithm. Any parameters of interest which have an STi that is less than or equal to the dummy parameter is considered not significantly different from zero. Comparisons between each parameter of interest and that of the dummy parameter are made using a two-sample t-test. This leads to the calculation of a p-value for the sensitivity indexes, which can also be seen in the summary spreadsheet. Thus in our example, there are four measures which are significant in terms of affecting a cells velocity (thresholdBindProbability, chemoThreshold, chemoLowerLinearAdjust, maxVCAMeffectProbabilityCutoff).

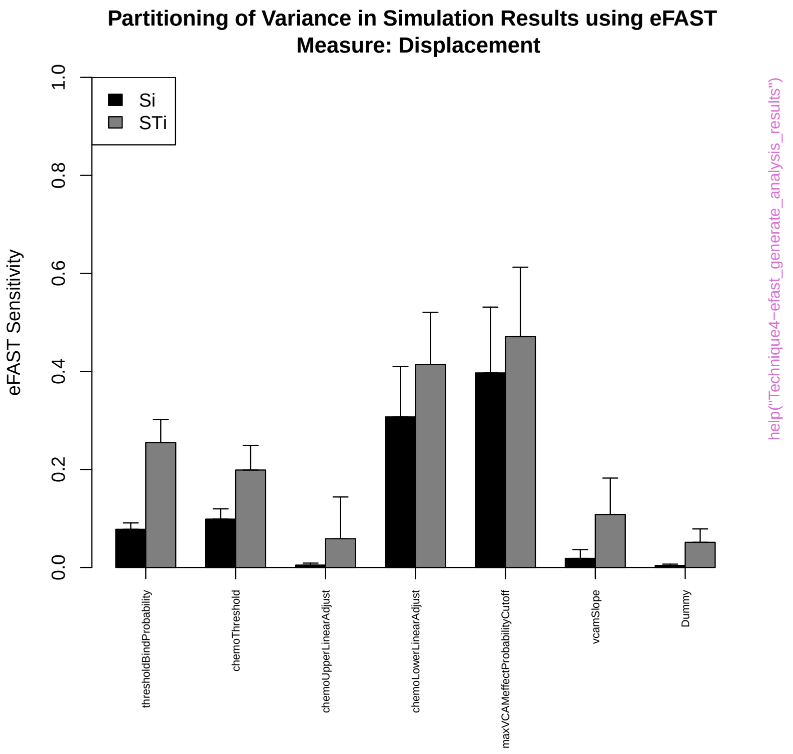

Figure 2: Graph showing the partitioning of simulation output variance between the input parameters, for the cell displacement measure. Si - Variance accounted for by that parameter; STi - Variance accounted for by its complement set

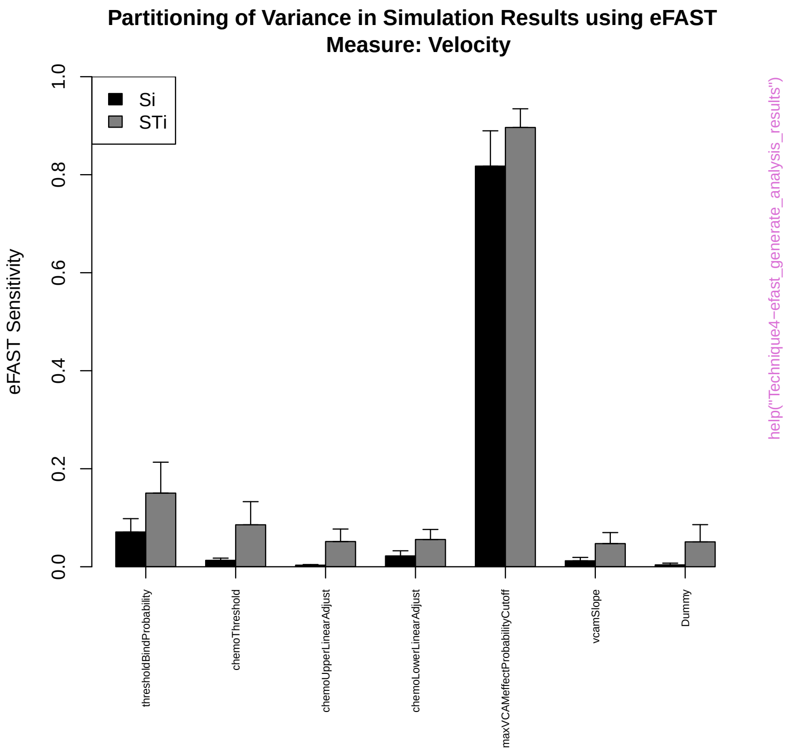

Figure 3: Graph showing the partitioning of simulation output variance between the input parameters, for the cell velocity measure. Si - Variance accounted for by that parameter; STi - Variance accounted for by its complement set

9 Running eFAST Technique for Multiple Timepoints

The package also has the capability to perform the above analysis for simulation results taken at different timepoints. This may give an indication of when trends tend to emerge, or certain parameters become influential. Again, we will examine this with an example, yet there is not much to change from the example seen previously

In this case study, we have captured the cell behaviour measures at multiple timepoints in the simulation, specifically 12, 36, 48,and 60 hours. Thus we have the output files trackedCells_Close_12.csv, trackedCells_Close_36.csv etc. To use this method over multiple timepoints, you should have (a) the same folder structure as in the previous example, and (b) an output file for each timepoint, with the timepoint appended to the filename after an underscore. It is worth writing a script to put your output in this format before looking at this method.

We explain how this works through an example, which adapts what we did previously.

1. Open the R script that you generated above in a text editor.

2. We now need to change the value of two of the R variables. These are:

   ```
   TIMEPOINTS, TIMEPOINTSCALE
   ```

   Change the values so these match the below:

   ```
   TIMEPOINTS<-c(12,36,48,60)
   TIMEPOINTSCALE<-"Hours"
   ```

   This alteration sets TIMEPOINTS to an array of timepoints being analysed, and TIMEPOINTSCALE to a string stating what these timepoints represent. The latter is used for graphing results in the final part of the method.

   Each R method will now process each timepoint in turn and append the timepoint being analysed onto the input and output file names, so that distinct results are output for each. For example, when the 12 hour timepoint is being examined, the file addresses will become trackedCells_Close_12.csv, EgSet_Medians_12.csv, etc.

3. In the text editor, delete the three R methods used previously (under the variable declarations) and add these methods in their place:

   ```
   #efast_generate_medians_for_all_parameter_subsets_overTime(FILEPATH,
   # NUMCURVES,PARAMETERS,NUMSAMPLES,NUMRUNSPERSAMPLE,MEASURES,
   # RESULTFILEFORMAT,RESULTFILENAME,ALTERNATIVEFILENAME,
   # OUTPUTCOLSTART,OUTPUTCOLEND,MEDIANSFILEFORMAT,MEDIANSFILENAME,
   # TIMEPOINTS)

   efast_get_overall_medians_overTime(FILEPATH,NUMCURVES,PARAMETERS,
   NUMSAMPLES,MEASURES,MEDIANSFILEFORMAT,MEDIANSFILENAME,
   CURVERESULTSFILENAME,TIMEPOINTS)

   efast_run_Analysis_overTime(FILEPATH,MEASURES,PARAMETERS,
   NUMCURVES,NUMSAMPLES,CURVERESULTSFILENAME,EFASTRESULTFILENAME,
   OUTPUTMEASURES_TO_TTEST,TTEST_CONF_INT,GRAPH_FLAG,
   TIMEPOINTS,TIMEPOINTSCALE)
   ```

Note that the first is highlighted out on this occasion as the simulation data in the download is not complete due to download file restrictions. The subtle change you will notice is that TIMEPOINTS and TIMEPOINTSCALE are now added to the top two methods. When each method is called, the method goes through each timepoint in turn. It will prepare the input and output filenames as stated above (adding the timepoint), then uses the same method as used in our first example (where only the end time point was analysed). Thus, whereas the first example produced output for one timepoint, R will now generate the same but for a number of different timepoints. To note the timepoint that was analysed, the graphs will have the timepoint appended to the filename in the same way as described previously.

4. Save the script in the text editor and run in R. Notice that results similar to those described previously are produced, but this time for all the timepoints specified.

10 Further Reading

The following references may be useful in understanding this technique in more detail:

- Read, M., Andrews, P.S., Timmis, J. & Kumar, V. (2012) Techniques for Grounding Agent-Based Simulations in the Real Domain : a case study in Experimental Autoimmune Encephalomyelitis. Mathematical and Computer Modelling of Dynamical Systems, 18(1):67-86.
- Marino, S., Hogue, I.B., Ray, C.J. & Kirschner, D.E. (2008) A methodology for performing global uncertainty and sensitivity analysis in systems biology. Journal of theoretical biology, 254 (1), p.pp.178-96.
- Saltelli, A., Chan, K. & Scott, E.M. (2000) Sensitivity Analysis, Wiley series in probability and statistics Wiley.
- The spartan Manual, spartan-Manual.pdf, within the spartan package describes in more detail each method within the package
