# Supplementary material for: Spartan: A Comprehensive Tool for Understanding Uncertainty in Simulations of Biological Systems
Source: PLoS Comput Biol. 2013 Feb 28;9(2):e1002916. doi: 10.1371/journal.pcbi.1002916 (PMC3585389; doi:10.1371/journal.pcbi.1002916)
Supplement: Software S1 — Spartan R package for Linux and Mac OS. Includes tutorials for each technique. (ZIP) [file pcbi.1002916.s001.zip › Technique3_Tutorial.pdf]

Tutorial on using the *spartan* package to analyse agent-based simulation results

For use with *spartan* package version 1.2 onwards

Technique 3: Parameter Sampling and Analysis through use of a Latin-Hypercube approach

1 Introduction

spartan, or (**S**imulation **P**arameter **A**nalysis **R** **T**oolkit **A**pplicatio**N**) is an R package which aids the understanding of the effect aleatory and epistemic uncertainty have on the output from a simulation. This set of tutorials makes use of available example simulation output to demonstrate how a variety of methods can be applied to further understand the results that have been generated. Following through each example should make it easier to apply the tookit to results generated by any agent-based computer simulation. This tutorial focuses on the identification of any non-linear effects which occur when two or more parameters are adjusted simultaneously.

2 The *spartan* Package

Computer simulations are becoming a popular technique to use in attempts to further our understanding of complex systems. This package provides code for four techniques described in available literature which aid the analysis of simulation results, at both single and multiple timepoints in the simulation run. The first technique addresses aleatory uncertainty in the system caused through inherent stochasticity, and determines the number of replicate runs necessary to generate a representative result. The second examines how robust a simulation is to parameter perturbation, through the use of a one-at-a-time parameter analysis technique. Thirdly, a latin hypercube based sensitivity analysis technique is included which can elucidate non-linear effects between parameters and indicate implications of epistemic uncertainty with reference to the system being modelled. Finally, a further sensitivity analysis technique, the extended Fourier Amplitude Sampling Test (eFAST) has been included to partition the variance in simulation results between input parameters, to determine the parameters which have a significant effect on simulation behaviour.

3 The Case Study

The example simulation results have been taken from an ongoing project which seeks to understand the formation of lymphoid tissue in the small intestine. This simulation outputs cell behaviour measures at various points in the simulation and measures describing the development of the tissue, which occurs through interactions between the cells. Techniques 2-4 of this package allow us to explore how input parameter value affects the behaviour of these cells. We need Technique 1 to tell us how many simulation runs we need for each condition explored to ensure we have a robust representative result.

4 Scope

Do note that the idea of this tutorial is to demonstrate the application of the toolkit, and is not intended to act as a full introduction to using Sensitivity Analysis techniques in the analysis of simulation results. Where useful, links to further reading have been included.

5 Prerequisites

- The R statistical environment, version 2.13.1 or later.
- The spartan R package, downloaded from the Comprehensive R Archive Network (CRAN) or from the project website.
- The lhs and gplots R packages, available for download from CRAN.
- The example simulation data for this tutorial, available from the project website.
- From version 1.2 of *spartan*, simulation results can be in either CSV or XML format. For earlier versions, results must be pre-processed to be in CSV format.

6 Running Technique 3: Latin-Hypercube Sampling and Analysis

Though Technique 2 of this toolkit elucidates the effects of perturbations of one parameter, it cannot show any non-linear effects which occur when two or more are adjusted simultaneously. A Global Sensitivity Analysis technique is needed to identify such effects, and to give an indication of the parameters which have the greatest influence on the simulation output. Thus we have included this method described by Read et al, Saltelli et al, and others (References at the end of this document). A set of parameters of interest are identified, and a range of potential values each parameter could lie within is assigned. A number of parameter value sets are then created through a latin-hypercube sampling approach, which selects values for each parameter from the parameter space, while aiming to reduce any possible correlations when the sample is produced. A number of simulation runs are then performed for each set generated (this number that which has become apparent through analysis of aleatory uncertainty, or use of Technique 1 within the *spartan* package). The simulation results are then analysed in such a way that any non-linear effects can be identified with ease, both visually and through the calculation of a Partial Rank Correlation Coefficient. The explanation of this is greatly aided by an example, which is covered later in this tutorial.

The *spartan* package includes methods to both create parameter value samples using the latin-hypercube approach, and to analyse the simulation results. This tutorial covers both methods.

7 Parameter Sampling

The package contains a method which can produce parameter value sets using Latin-Hypercube sampling. Simulations should then be run on each of the generated parameter sets. Sampling is performed as follows:

1. Open a text editor (gedit or similar). Now we are going to declare the variables required by the package to produce the parameter value sets. Type or copy in the text on Page 3. Firstly, the *spartan* library is imported. The R variables required for this analysis are then declared in capital letters. The line underneath each, beginning with a #, is a description

of that being declared. Make sure you set the FILEPATH variable correctly to match the folder where you would like the parameter value sets to be output to.
The last variable, ALGORITHM, controls whether a fully optimised latin-hypercube algorithm is used, or parameter values chosen from each section of the hypercube randomly. Both these algorithms are taken from the lhs package. Note that although an optimised sample may be preferable, the generation of parameter values using an optimal algorithm may take a long time (in our experience, over 24 hours for just 7 parameters). The ALGORITHM variable can be set to either "normal" or "optimal"

```
library(spartan)
# Import the package

FILEPATH<-"/media/FreeAgent/package_Test_Data/LHC/Sampling"
# The folder where the results should be output to
PARAMETERS <- c("thresholdBindProbability","chemoThreshold",
"chemoUpperLinearAdjust","chemoLowerLinearAdjust",
"maxVCAMeffectProbabilityCutoff","vcamSlope")
# Names of the parameters to generate parameter value samples for.
# Should be within an array
NUMSAMPLES <- 65
# The number of parameter sample sets to create using the hypercube
PMIN<-c(0,0.10,0.10,0.015,0.1,0.25)
# The minimum value in the range for each parameter
PMAX<-c(100,0.9,0.50,0.08,1.0,5.0)
# The maximum value in the range for each parameter
ALGORITHM<-"normal"
# Algorithm to use to generate the hypercube
```

2. To get the value sets for each parameter, the following method is used. Copy the text below into the R script file below the declarations you have made above. Note that the first line imports the lhs library. You should have already installed this package into your R environment as noted in the preqrequisites for this tutorial.

```
library(lhs)
lhc_generate_lhc_sample(FILEPATH,PARAMETERS,NUMSAMPLES,PMIN,PMAX,ALGORITHM)
```

3. Save the file with a suitable filename and .R extension (for example LHC_Sampling.R)

4. For Linux or Mac OS, open a command prompt, and navigate to the directory where this file was saved. Type the following:

```
Rscript LHC_Sampling.R
```

If you are using Windows, open R and type the following into the R Command Prompt (where [*path to directory* is the full path to where LHC_Sampling.R is saved]:

```
source("C:\[\textit{path to directory}]\LHC_Sampling.R")
```

This will produce a CSV file containing, in this case, 65 sets of parameter values generated using the hypercube. This CSV file will be stored in the FILEPATH specified as LHC_Parameters_for_Runs.csv. Simulations should then be run on each parameter value set in each file, and analysed using the technique in the next part of this tutorial.

8 Parameter Analysis

This section of the tutorial performs this analysis for the lymphoid tissue formation simulation. In this case, we are going to examine two cell behaviour measures, Velocity and Displacement, that are captured for a period representing one hour of real time, to determine how a change in parameter value influences this behaviour.

The assumption is made that for each set of parameters generated by the hypercube, a number of replicate simulation runs have been performed to produce a robust result. This number is elucidated using Technique 1 of this toolkit. So in the example case we are going to use here, we generated 500 different parameter value sets, and for each we have performed 300 simulation runs. The algorithm takes each parameter set generated in turn, processing the simulation results to generate median values for each output measure, for each of the 300 runs. Thus for each of the 500 sets of values, a file is created which shows the median of each output measure for each of the 300 runs. This file can be in either XML or CSV format. A summary file is then created, containing the values of each parameter in that set alongside the median of the 300 medians. An example of such a file can be seen in the data directory of the package (EgSet_LHC_Summary.csv). With this summary complete, each parameter being analysed is processed in turn, to determine if there are any correlations between the value of this parameter and simulation output result, although all other parameter values have been perturbed. Partial Rank Correlation Coefficients (PRCC) are generated for each output measure, for each parameter. These give a statistical indication of any correlations that have now become apparent, and are recorded in a CSV file. An example of this can again be seen in the data directory of the package (EgSet_LHC_corCoeffs.csv). To ease identification of such effects, graphs are produced for each parameter, showing the parameter value against the simulation result (output measure).

An example will make this clearer.

1. Download the LHC example data from the project website and extract the results.
2. The first thing to note is the folder structure. To use this method, the simulation results do need to be in a specific format (Figure 1 LHC folder structure). The structure has two levels:
   (i) Folders for each set of parameter values being analysed. In the example case, the parameter space was sampled 500 times, thus there are 500 folders, numbered 1-500.
   (ii) A folder for each of the simulation results where the simulation was run under those conditions. Will match the number of runs required that was determined through Aleatory Analysis (Technique 1). So, if this was 300, there would be 300 folders numbered 1-300
3. With this data available, open a text editor (gedit or similar). Now we are going to declare the variables required to run this analysis. Type or copy in the text on Page 6. Firstly, the *spartan* library is imported. The R variables required for this analysis are then declared in capital letters. The line underneath, beginning with a #, is a description of that being declared. Make sure you set the FILEPATH variable correctly to state where the simulation results have been extracted to.

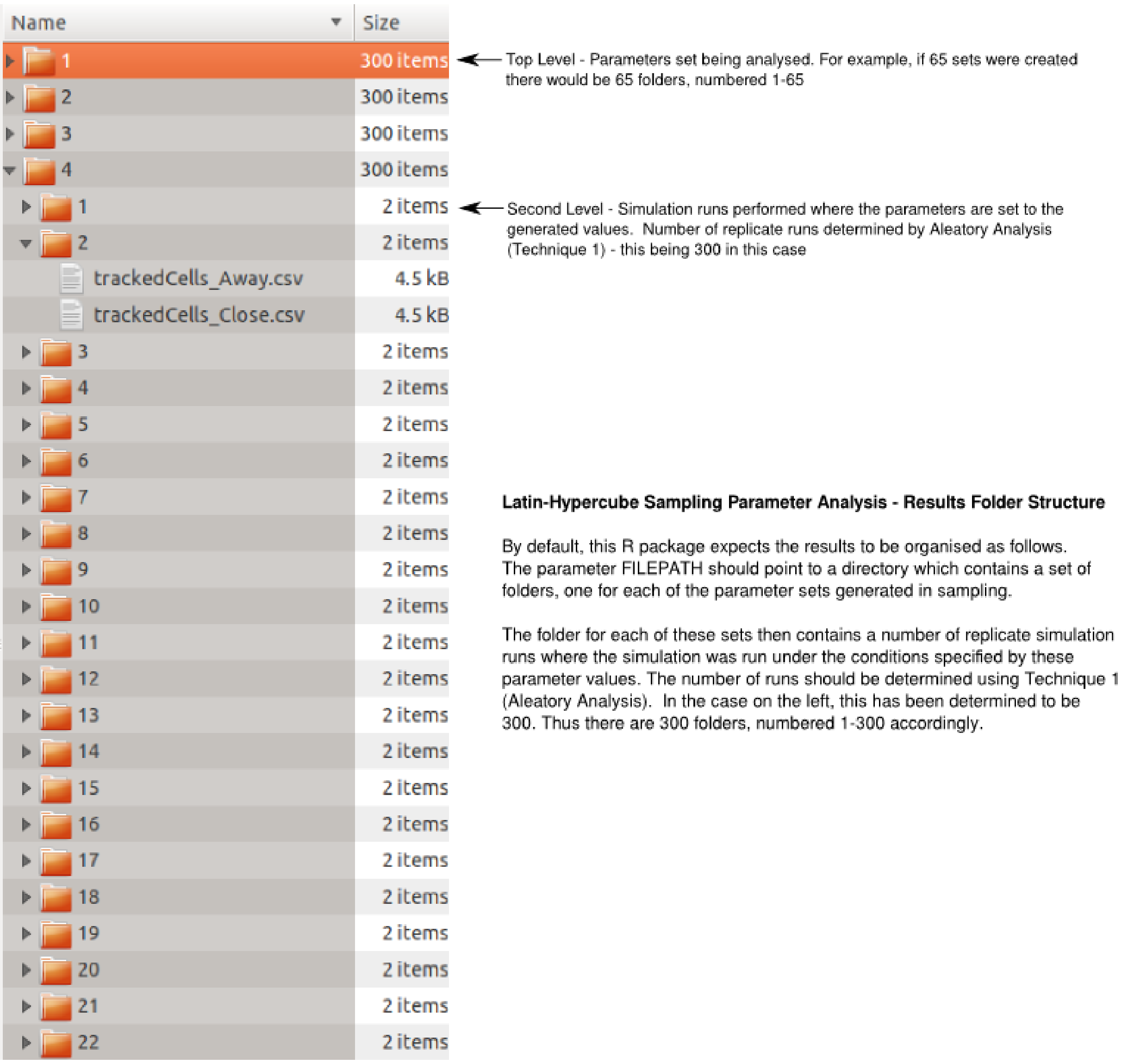

Figure 1: Simulation results folder structure that should exist for use with this tool

```
library(spartan)
# Import the package

FILEPATH<-"/media/FreeAgent/package_Test_Data/LHC/LHC_Results"
# Folder containing the simulation results
PARAMETERS<-c("thresholdBindProbability","chemoThreshold",
"chemoUpperLinearAdjust","chemoLowerLinearAdjust",
"maxVCAMeffectProbabilityCutoff","vcamSlope")
# Array of the parameters to be analysed
MEASURES<-c("Velocity","Displacement")
# The simulation output measures being examined
MEASURE_SCALE<-c("microns/min","microns")
# What each measure represents.  Used in graphing results
NUMSAMPLES <- 500
# The number of parameter value sets created in latin-hypercube
# sampling
NUMRUNSPERSAMPLE<-300
# Number of runs performed for each parameter value set
RESULTFILEFORMAT<-"csv"
# Simulation result file format. Should be either CSV or XML
RESULTSFILENAME<-"trackedCells_Close"
# The output file containing the simulation results from that simulation run
# Note no file extension
ALTERNATIVEFILENAME<-NULL
# Not used in this case, but this is useful in cases where two result files may
# exist (for example if tracking cells close to an area, and those further away
#  two output files could be used).  Here, results in a second file are processed
# if the first is blank or does not exist. Note no file extension if used.
OUTPUTCOLSTART<-10
# Use this if simulation results are in CSV format.
# The column within the csv results file where the results start.  This is useful
# as it restricts what is read in to R, getting round potential errors where the
# first column contains an agent label (as R does not read in CSV files where the
# first column contains duplicates)
OUTPUTCOLEND<-11
# Use this if simulation results are in CSV format.
# Last column of the output measure results
MEDIANSFILEFORMAT<-"csv"
# File format of median results file that will be generated in this tutorial.
# Should be either XML or CSV. This is used as, for some applications, a simulation
# results set for processing may have been generated using methods other than
# spartan, and may not be in CSV format.
MEDIANSFILENAME<-"EgSet_Medians"
# For each parameter value set being analysed, a file is created containing the
# median of each output measure, of each simulation run for that value. This sets
# the name of this file. Note no file extension
LHC_PARAM_CSV_LOCATION<-
"/media/FreeAgent/package_Test_Data/LHC/LHC_Results/LHC_Parameters_for_Runs.csv"
# Location of a file containing the parameter value sets generated by the
# hypercube sampling (i.e. the file generated in the previous method of this
# tutorial. Unlike above, an extension should be specified.
LHCSUMMARYFILENAME<-"EgSet_LHCSummary"
```

```
# File name to give to the summary file that is produced showing the parameter value
# sets alongside the median results for each simulation output measure. Note no
# file extension
CORCOEFFSOUTPUTFILE<-"EgSet_corCoeffs"
# File name to give to the file showing the Partial Rank Correlation Coefficients
# for each parameter. Again note no file extension
TIMEPOINTS<-NULL; TIMEPOINTSCALE<-NULL
# Not used in this case, but when a simulation is analysed at multiple timepoints
# (see later in tutorial)
```

4. Now to examine the first of the four methods (we are going to do each individually in the tutorial so the functionality becomes apparent but in reality you will run all three methods one after another in the same text file). Copy the below into the text file under the above declarations:

```
lhc_process_sample_run_subsets(FILEPATH,NUMSAMPLES,
NUMRUNSPERSAMPLE,MEASURES,RESULTFILEFORMAT,
RESULTSFILENAME,ALTERNATIVEFILENAME,OUTPUTCOLSTART,
OUTPUTCOLEND,MEDIANSFILEFORMAT,MEDIANSFILENAME)
```

   This takes each parameter value set generated by the hypercube in turn, and analyses the simulation results. For each parameter set, there should be n simulation results. This method goes through these results, producing a file containing the median of each output measure for each of the n runs. Thus, if n=300, as in our example case, the median file will contain 300 medians for each simulation output measure. The median file generated will be stored with the file name given in MEDIANSFILENAME, in the format specified by MEDIANSFILEFORMAT

   In normal cases, you would now save the text file and run the script in R to generate these files. However, unlike the tutorials on Technique 1 and 2, actual simulation results have not been included in the download due to the excessive file such from such a large number of simulations runs. Instead, the data included is the output of the above method once run. Have a look at one of the files to ensure you understand the format, but do not run this method else you will get an error in this case. Instead, change the text file so these lines are commented out, like this:

```
#lhc_process_sample_run_subsets(FILEPATH,NUMSAMPLES,
# NUMRUNSPERSAMPLE,MEASURES,RESULTFILEFORMAT,
# RESULTSFILENAME,ALTERNATIVEFILENAME,OUTPUTCOLSTART,
# OUTPUTCOLEND,MEDIANSFILEFORMAT,MEDIANSFILENAME)
```

5. We are now going to run the second method, which takes the set of medians contained in each of the parameter value set folders and produces a file summarising the simulation results. In the same file in the text editor, add the following method call, then save the file.

```
lhc_generateLHCSummary(FILEPATH,LHC_PARAM_CSV_LOCATION,
PARAMETERS,NUMSAMPLES,MEASURES,MEDIANSFILEFORMAT,
MEDIANSFILENAME,LHCSUMMARYFILENAME)
```

   This again goes through each parameter value set in turn, but this time looks at the medians for each run (stored within the file stated by MEDIANFILENAME). The median of these medians, for each simulation output measure, is calculated. The method then reads in the values that were assigned for each parameter in that run from the sampling result CSV file stated in LHC_PARAM_CSV_LOCATION (as was generated during sampling) and stores the median simulation results alongside the parameter values that were assigned. Once this is completed for all parameter value sets, this is output as a CSV file, filename specified in LHCSUMMARYFILENAME.

6. For Linux or Windows OS, open a command prompt, and navigate to the directory where this file was saved. Type the following:

```
Rscript LHC_Analysis.R
```

If you are using Windows, open R and type the following into the R Command Prompt (where [*path to directory* is the full path to where LHC_Analysis.R is saved]:

```
source("C:\[\textit{path to directory}]\LHC_Analysis.R")
```

Navigate through the folder structure and make yourself familiar with the format of the file produced. This will help when applying the toolkit to other simulations.

7. With this summary produced, it is now possible to elucidate any non-linear effects that are apparent when all parameter values are perturbed concurrently. To get a statistical measure of the presence of any effects, the Partial Rank Correlation Coefficient is generated for each parameter. This examines each parameter in turn and each simulation output value, considering the value it has been assigned against the simulation result. The result of the calculation for each parameter is output to a CSV file, with filename as specified by CORCOEFFSOUTPUTFILE.

   In the same file in the text editor, add the following method call, then save the file.

   ```
   lhc_generatePRCoEffs(FILEPATH,PARAMETERS,MEASURES,
   LHCSUMMARYFILENAME,CORCOEFFSOUTPUTFILE)
   ```

8. Return to the command prompt and run the R script using the same command as previously. This will then generate the Partial Rank Correlation Coefficients results file, within the folder specified by FILEPATH. Navigate to the folder and make sure you are familiar with the structure of this file.

9. Go back to the text editor. We are now going to represent the results graphically, making any effects present easier to notice. Add the following to the script text file:

   ```
   lhc_graphMeasuresForParameterChange(FILEPATH,PARAMETERS,
   MEASURES,MEASURE_SCALE,CORCOEFFSOUTPUTFILE,
   LHCSUMMARYFILENAME,TIMEPOINTS,TIMEPOINTSCALE)
   ```

10. Return to the command prompt and run the R script using the same command as previously.

    This produces a graph for each parameter of interest, and each simulation output measure for that parameter, plotting the value that parameter was assigned against the simulation result. This makes trends easy to identify. For each graph, the respective Partial Rank Correlation Coeffient is included in the title to give a statistical measure to the results seen visually. Two of the graphs that were generated can be seen in Figure 2. Note how for the first parameter, which captures cell adhesion, there is a definite correlation between the Velocity simulation output measure and the value of that parameter, although all other parameters of interest were also being perturbed at the same time. For the second, which captures the effect of a chemoattractant, note that no such trend has emerged. This gives the suggestion that the value of the first parameter is highly influential in affecting simulation behaviour.

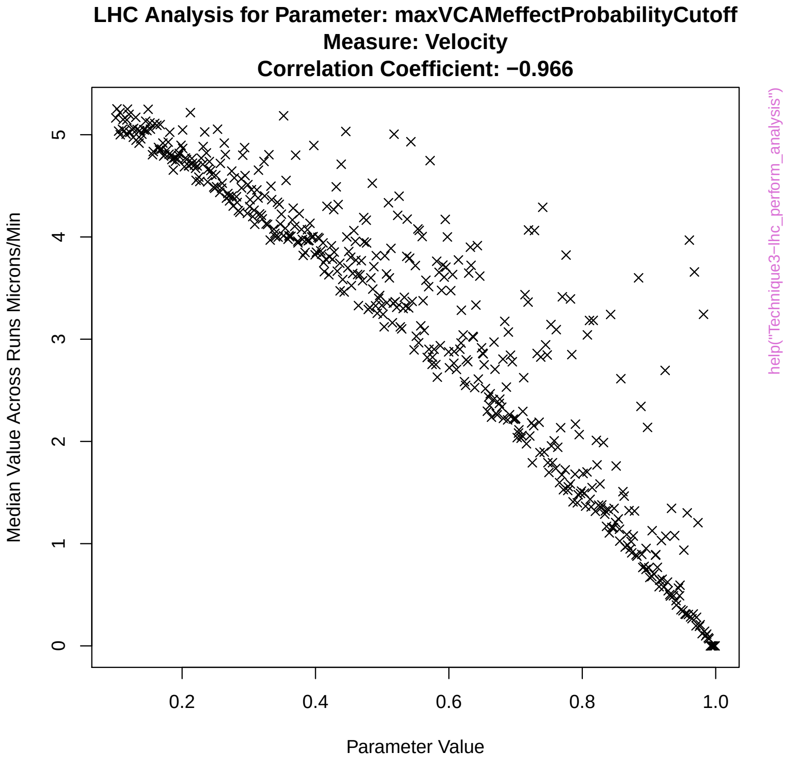

Figure 2: Graph showing the median velocity observed when the adhesion factor parameter value is perturbed

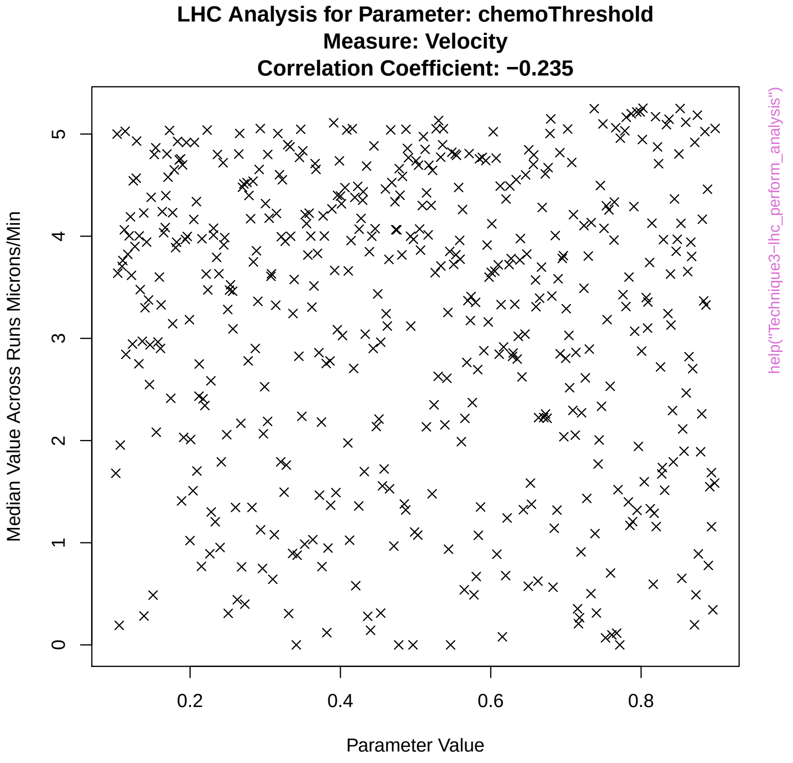

Figure 3: Graph showing the median velocity observed when the chemoattractant parameter value is perturbed

9 Running LHC Technique for Multiple Timepoints

The package also has the capability to perform the above analysis for simulation results taken at different timepoints. This may give an indication of when such trends tend to emerge. Again, we will examine this with an example, yet there is not much to change from the example seen previously

In this case study, we have captured the cell behaviour measures at multiple timepoints in the simulation, specifically 12, 36, 48,and 60 hours. Thus we have the output files trackedCells_Close_12.csv, trackedCells_Close_36.csv etc. To use this method over multiple timepoints, you should have (a) the same folder structure as in the previous example, and (b) an output file for each timepoint, with the timepoint appended to the filename after an underscore. It is worth writing a script to put your output in this format before looking at this method.

We explain how this works through an example, which adapts what we did previously.

1. Open the R script that you generated above in a text editor.
2. We now need to change the value of some of the variables. These are:

   ```
   TIMEPOINTS, TIMEPOINTSCALE
   ```

   Change the values so they match these below:

   ```
   TIMEPOINTS<-c(12,36,48,60)
   TIMEPOINTSCALE<-"Hours"
   ```

   This sets TIMEPOINTS to an array of timepoints being analysed, and TIMEPOINTSCALE to a string stating what these timepoints represent. The latter is used for graphing results in the final part of the method.

   Each R method will now process each timepoint in turn and append the timepoint being analysed onto the input and output file names, so that distinct results are output for each. For example, when the 12 hour timepoint is being examined, the file addresses will become trackedCells_Close_12.csv, EgSet_Medians_12.csv, etc.

3. In the text editor, delete the four R methods used previously (under the parameter declarations) and add these methods in their place:

   ```
   #lhc_process_sample_run_subsets_overTime(FILEPATH,
   # NUMSAMPLES,NUMRUNSPERSAMPLE,MEASURES,
   # RESULTFILEFORMAT,RESULTFILENAME,ALTERNATIVEFILENAME,
   # OUTPUTCOLSTART,OUTPUTCOLEND,MEDIANSFILEFORMAT,
   # MEDIANSFILENAME,TIMEPOINTS)

   lhc_generateLHCSummary_overTime(FILEPATH,
   LHC_PARAM_CSV_LOCATION,PARAMETERS,NUMSAMPLES,
   MEASURES,MEDIANSFILEFORMAT,MEDIANSFILENAME,
   LHCSUMMARYFILENAME,TIMEPOINTS)

   lhc_generatePRCoEffs_overTime(FILEPATH,
   PARAMETERS,MEASURES,LHCSUMMARYFILENAME,
   CORCOEFFSOUTPUTFILE,TIMEPOINTS)
   ```

```
lhc_graphMeasuresForParameterChange_overTime(
FILEPATH,PARAMETERS,MEASURES,MEASURE_SCALE,
LHCSUMMARYFILENAME,CORCOEFFSOUTPUTFILE,TIMEPOINTS,
TIMEPOINTSCALE)
```

Again, note the first is highlighted out as the full simulation data is not included in the download due to file size restrictions. The subtle change you will notice is that TIMEPOINTS and TIMEPOINTSCALE are now added to the top two methods. When each method is called, the method goes through each timepoint in turn. It will prepare the input and output filenames as stated above (adding the timepoint), then uses the same method as used in our first example (where only the end time point was analysed). Thus, whereas the first example produced output for one timepoint, R will now generate the same but for a number of different timepoints. To note the timepoint that was analysed, the graphs will have the timepoint appended to the filename in the same way as described previously.

4. Save the script in the text editor and run in R. Notice that results similar to those described previously are produced, but this time for all the timepoints specified.

10 Further Reading

The following references may be useful in understanding this technique in more detail:

- Read, M., Andrews, P.S., Timmis, J. & Kumar, V. (2012) Techniques for Grounding Agent-Based Simulations in the Real Domain : a case study in Experimental Autoimmune Encephalomyelitis. Mathematical and Computer Modelling of Dynamical Systems, 18(1):67-86.
- Marino, S., Hogue, I.B., Ray, C.J. & Kirschner, D.E. (2008) A methodology for performing global uncertainty and sensitivity analysis in systems biology. Journal of theoretical biology, 254 (1), p.pp.178-96.
- Saltelli, A., Chan, K. & Scott, E.M. (2000) Sensitivity Analysis, Wiley series in probability and statistics Wiley.
- The *spartan* Manual, spartan-Manual.pdf, within the spartan package describes in more detail each method within the package
