# Supplementary material for: Spartan: A Comprehensive Tool for Understanding Uncertainty in Simulations of Biological Systems
Source: PLoS Comput Biol. 2013 Feb 28;9(2):e1002916. doi: 10.1371/journal.pcbi.1002916 (PMC3585389; doi:10.1371/journal.pcbi.1002916)
Supplement: Software S1 — Spartan R package for Linux and Mac OS. Includes tutorials for each technique. (ZIP) [file pcbi.1002916.s001.zip › Technique2_Tutorial.pdf]

Tutorial on using the *spartan* package to analyse agent-based simulation results

For use with *spartan* package version 1.2 onwards

Technique 2: One-A-Time Parameter Analysis

1 Introduction

spartan, or (**S**imulation **P**arameter **A**nalysis **R** **T**oolkit **A**pplicatio**N**) is an R package which aids the understanding of the effect aleatory and epistemic uncertainty have on the output from a simulation. This set of tutorials makes use of available example simulation output to demonstrate how a variety of methods can be applied to further understand the results that have been generated. Following through each example should make it easier to apply the tookit to results generated by any agent-based computer simulation. This tutorial focuses on understanding how robust the behaviour of a simulator is to parameter perturbtation.

2 The *spartan* Package

Computer simulations are becoming a popular technique to use in attempts to further our understanding of complex systems. This package provides code for four techniques described in available literature which aid the analysis of simulation results, at both single and multiple timepoints in the simulation run. The first technique addresses aleatory uncertainty in the system caused through inherent stochasticity, and determines the number of replicate runs necessary to generate a representative result. The second examines how robust a simulation is to parameter perturbation, through the use of a one-at-a-time parameter analysis technique. Thirdly, a latin hypercube based sensitivity analysis technique is included which can elucidate non-linear effects between parameters and indicate implications of epistemic uncertainty with reference to the system being modelled. Finally, a further sensitivity analysis technique, the extended Fourier Amplitude Sampling Test (eFAST) has been included to partition the variance in simulation results between input parameters, to determine the parameters which have a significant effect on simulation behaviour.

3 The Case Study

The example simulation results have been taken from an ongoing project which seeks to understand the formation of lymphoid tissue in the small intestine. This simulation outputs cell behaviour measures at various points in the simulation and measures describing the development of the tissue, which occurs through interactions between these cells. Techniques 2-4 of this package allow us to explore how input parameter value affects the behaviour of these cells. We need Technique 1 to tell us how many simulation runs we need for each condition explored to ensure we have a robust representative result.

4 Scope

Do note that the idea of this tutorial is to demonstrate the application of the toolkit, and is not intended to act as a full introduction to using Sensitivity Analysis techniques in the analysis of simulation results. Where useful, links to further reading have been included.

5 Prerequisites

- The R statistical environment, version 2.13.1 or later.
- The spartan R package, downloaded from the Comprehensive R Archive Network (CRAN) or from the project website.
- The lhs and gplots R packages, available for download from CRAN.
- The example simulation results, available from the project website.
- From version 1.2 of *spartan*, simulation results can be in either CSV or XML format. For earlier versions, results must be pre-processed to be in CSV format.

6 Running Technique 2: One-A-Time (OAT) Parameter Analysis

The robustness of a simulation to parameter alteration can be determined through the use of this approach. Following the method described by Read et al (2012), a set of parameters of interest are identified, and a range of potential values each parameter could lie within is assigned. The technique examines the sensitivity to a change in one parameter. Thus, the value of each is perturbed independently, with all other parameters remaining at their calibrated value. In this implementation, each parameter of interest is considered in turn. Initially the parameter is assigned the minimum value within its allocated range. A number of simulation runs are then performed under these conditions (this number that which has become apparent through analysis of aleatory uncertainty, or use of Technique 1 within the *spartan* package). The value of the parameter is then increased, and the runs repeated under this new criteria. This is repeated until the full range has been explored. For each value that the parameter was assigned, the results of each simulation are examined and the median values of the output measures calculated. This creates a set of median output values for each parameter value. This set is then compared to a set of median output measures where the parameter was set to its calibrated value, using the Vargha-Delaney A-Test (2000). This gives an indication of how different the two sets of results are, and thus the change in simulation behaviour when the parameter is perturbed.

The spartan package includes methods to both create parameter value samples and to analyse the simulation results. This tutorial covers both methods.

7 Parameter Sampling

The package contains a method which can produce a set of values for each parameter of interest. Simulations should then be run on each of the generated parameter sets. This is done as follows:

1. Open a text editor (gedit or similar). Now we are going to declare the variables required by the package to produce the parameter value sets. Type or copy in the text on Page 3. Firstly, the *spartan* library is imported. The variables required for this analysis are then declared in capital letters. The line underneath, beginning with a #, is a description of that being declared. Make sure you set the FILEPATH variable correctly to match the folder where you would like the parameter value sets to be output to.

```
library(spartan)
# Import the package

FILEPATH<-"/media/FreeAgent/package_Test_Data/OAT/Sampling"
# The folder where the results should be output to
PARAMETERS <- c("thresholdBindProbability","chemoThreshold",
"chemoUpperLinearAdjust","chemoLowerLinearAdjust",
"maxVCAMeffectProbabilityCutoff","vcamSlope")
# Names of the parameters to generate parameter value samples for.
# Should be within an array
BASELINE<-c(50,0.3,0.2,0.04,0.60,1.0)
# The calibrated values, or baseline values, for each parameter
PMIN<-c(0,0.10,0.10,0.015,0.1,0.25)
# The minimum value in the range for each parameter
PMAX<-c(100,0.9,0.50,0.08,1.0,5.0)
# The maximum value in the range for each parameter
PINC<-c(10,0.1,0.05,0.005,0.05,0.25)
# The increment to apply for each parameter when exploring the
# value range
```

2. To get the value sets for each parameter, the following method is used. Copy the text below into the R script file below the declarations you have made above:

   ```
   oat_parameter_sampling(FILEPATH,PARAMETERS,BASELINE,PMIN,PMAX,
   PINC)
   ```

3. Save the file with a suitable filename and .R extension (for example OAT_Sampling.R)

4. If you are using Linux or Mac OS, open a command prompt, and navigate to the directory where this file was saved. Type the following:

   ```
   Rscript OAT_Sampling.R
   ```

   If you are using Windows, open R and type the following into the R Command Prompt (where [*path to directory* is the full path to where OAT_Sampling.R is saved]:

   ```
   source("C:\[\textit{path to directory}]\OAT_Sampling.R")
   ```

   This will produce a CSV for for each parameter (so in the example case, 6 are generated), with each having a name matching the parameter which is being perturbed. Simulations should then be run on each parameter value set in each file, and analysed using the technique in the next part of this tutorial.

8 Parameter Analysis

This section of the tutorial performs this analysis for the lymphoid tissue formation simulation. In this case, we are going to examine two cell behaviour measures, Velocity and Displacement, that are captured for a period representing one hour of real time, to determine how a change in parameter value influences this behaviour. In the simulation results folders that you have downloaded from the website, the file we are going to look at is trackedCells_Close_Endpoint.csv. Due to size restrictions, the example simulation results set we will analyse only examines two parameters, these being chemoLowerLinearAdjust and chemoUpperLinearAdjust. In the case study, these control expression of a chemical attractant molecule.

1. Download the OAT example result set from the project website and extract the results. **NB:** This is a large file, make sure it has completely downloaded else it will not extract

2. The first thing to note is the folder structure. To use this method, the simulation results do need to be in a specific format (Figure 1 OAT folder structure). The structure has three levels:

   (i) Folders for each parameter being analysed (given that parameter name)

   (ii) Folders for each value that the parameter has been assigned (again given that parameter value)

   (iii) A folder for each of the simulation results where the simulation was run under those conditions. Will match the number of runs required that was determined through Aleatory Analysis (Technique 1). So, if this was 300, there would be 300 folders numbered 1-300

3. With this data available, open a text editor (gedit or similar). Now we are going to declare the variables required to run this analysis. Type or copy in the text on Page 5. Firstly, the *spartan* library is imported. The variables required for this analysis are then declared in capital letters. The line underneath, beginning with a #, is a description of that being declared. Make sure you set the FILEPATH variable correctly to state where the simulation results have been extracted to.

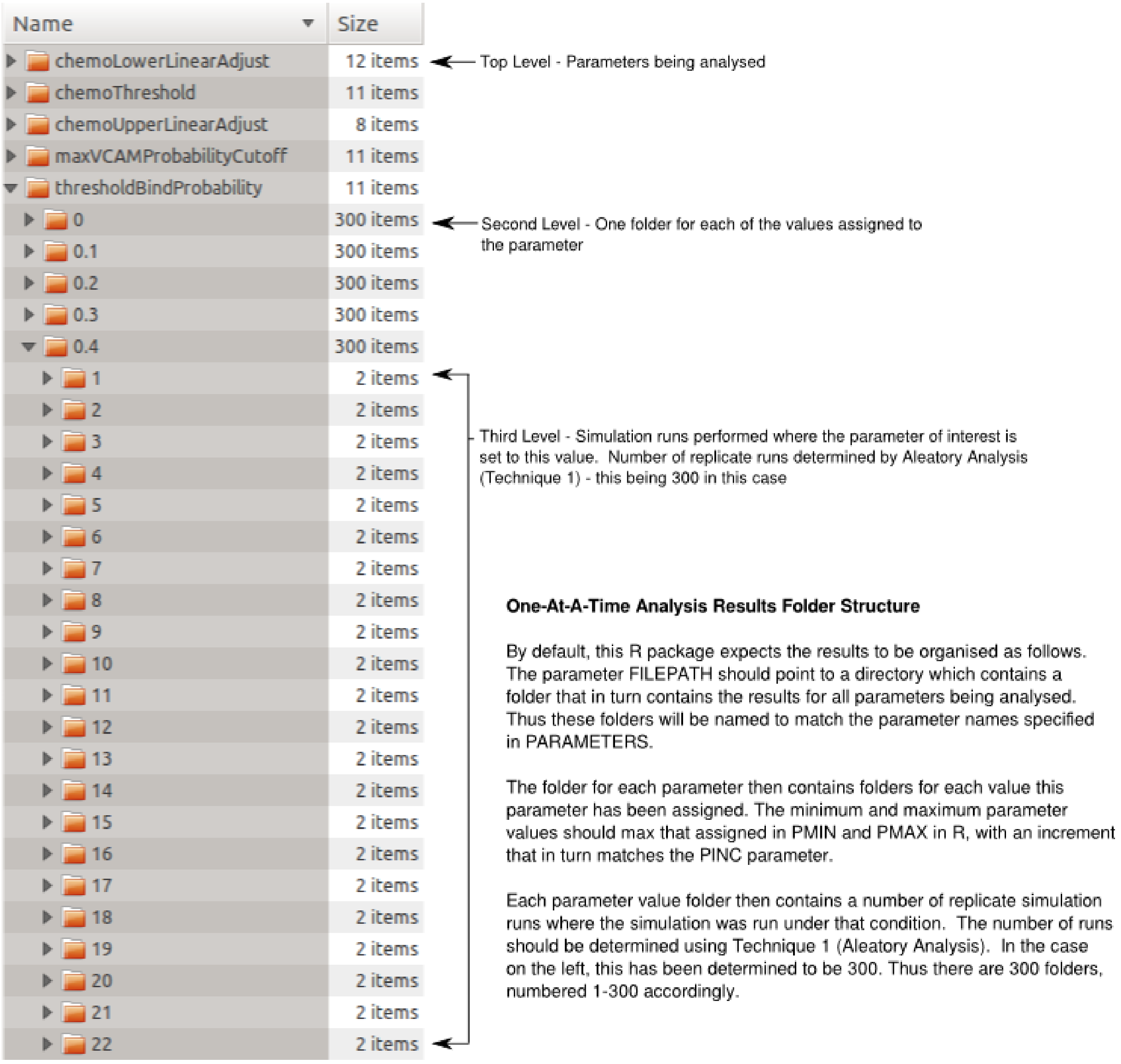

Figure 1: Simulation results folder structure that should exist for use with this tool

```
library(spartan)
# Import the package

FILEPATH<-"/media/FreeAgent/package_Test_Data/OAT"
# Folder containing the simulation results
PARAMETERS<-c("chemoLowerLinearAdjust","chemoUpperLinearAdjust")
# Array of the parameters to be analysed
PMIN<-c(0.015,0.10)
# The minimum value explored for each parameter
PMAX<-c(0.08,0.50)
# The maximum value for each parameter
PINC<-c(0.005,0.05)
# Amount the parameter value was incremened during sampling
BASELINE<-c(0.04,0.2)
# Value assigned to each parameter at calibration (the baseline value)
NUMRUNSPERSAMPLE<-300
# Number of runs performed for each parameter value set
MEASURES<-c("Velocity","Displacement")
# The simulation output measures being examined
RESULTFILEFORMAT<-"csv"
# Simulation result file format. Should be either CSV or XML
RESULTFILENAME<-"trackedCells_Close"
# The output file containing the simulation results from that simulation run.
# Note no file extension
OUTPUTCOLSTART<-10
# Use this if simulation results are in CSV format.
# The column within the csv results file where the results start.  This is useful
# as it restricts what is read in to R, getting round potential errors where the
# first column contains an agent label (as R does not read in CSV files where the
# first column contains duplicates)
OUTPUTCOLEND<-11
# Use this if simulation results are in CSV format.
# Last column of the output measure results
ALTERNATIVEFILENAME<-NULL
# Not used in this case, but this is useful in cases where two result files may
# exist (for example if tracking cells close to an area, and those further away
#  two output files could be used).  Here, results in a second file are processed
# if the first is blank or does not exist.
MEDIANSFILEFORMAT<-"csv"
# File format of median results file that will be generated in this tutorial.
# Should be either XML or CSV. This is used as, for some applications, a simulation
# results set for processing may have been generated using methods other than
# spartan, and may not be in CSV format.
MEDIANSFILENAME<-"EgSet_Medians"
# For each parameter value being analysed, a file is created containing the median
# of each output measure, of each simulation run for that value. This sets the
# name of this file. Note no file extension
ATESTRESULTSFILENAME<-"EgSet_ATests"
# The results of the A-Test comparisons of each parameter value against that of the
# parameters baseline value are output as a file. This sets the name of this file.
# Note no file extension. Current versions of spartan output to CSV files, though
# this may change in later versions.
```

```
ATESTSIGLEVEL<-0.21
# A-Test result value either side of 0.5 at which the difference between two sets of
# results is significant
MEASURE_SCALE<-c("microns/min","microns")
# What each measure represents.  Used in graphing results
TIMEPOINTS<-NULL; TIMEPOINTSCALE<-NULL
# Not used in this case, but when a simulation is analysed at multiple timepoints
# (see later in tutorial)
```

4. Now to run the first of the four methods (we are going to do each individually in the tutorial so the functionality becomes apparent but in reality you will run all three methods one after another in the same text file). Copy the below into the text file under the above declarations:

   ```
   oat_processParamSubsets(FILEPATH,PARAMETERS,PMIN,PMAX,PINC,
   NUMRUNSPERSAMPLE,MEASURES,RESULTFILEFORMAT,RESULTFILENAME,
   ALTERNATIVEFILENAME,OUTPUTCOLSTART,OUTPUTCOLEND,
   MEDIANSFILEFORMAT,MEDIANSFILENAME)
   ```

   This examines each parameter that is to be analysed in turn, starting with the minimum value it has been assigned and works through all values up to and including the maximum in the range. For each value, the median of each output measure is calculated for each run that has been performed, and these stored in a file within the folder for this parameter value (file given the name set in MEDIANSFILENAME). For example, if the parameter value is 0, and 300 runs have been performed where this is the case, a file is created containing the median of both the Velocity and Displacement output measure for each of the 300 runs. This will be in CSV or XML format, dependent on the format set by parameter MEDIANFILEFORMAT. This set of medians can then be compared with the known simulation behaviour at the baseline.

5. Save the file with a suitable filename and .R extension (for example OAT_Analysis.R)

6. For linux or Mac OS, open a command prompt, and navigate to the directory where this file was saved. Type the following:

   ```
   Rscript OAT_Analysis.R
   ```

   If you are using Windows, open R and type the following into the R Command Prompt (where [*path to directory* is the full path to where OAT_Analysis.R is saved]:

   ```
   source("C:\[\textit{path to directory}]\OAT_Analysis.R")
   ```

   Navigate through the folder structure and make yourself familiar with the format of the file produced. This will help when applying the toolkit to other simulations.

7. We are now going to run the second method, which gives an indication of the change in behaviour caused by this parameter value change. In the same file in the text editor, add the following method call, then save the file.

   ```
   oat_analyseAllParams(FILEPATH,PARAMETERS,BASELINE,PMIN,
   PMAX,PINC,MEASURES,MEDIANSFILEFORMAT,MEDIANSFILENAME,
   ATESTRESULTSFILENAME)
   ```

Again, this method examines each parameter in turn. The aim is to compare the median sets generated above with those obtained when the parameter was set to its calibrated, baseline, value. As an example, consider one of the parameters we are analysing in the example set, chemoLowerLinearAdjust. This has a baseline value of 0.04. The simulation has two output measures, Velocity and Displacement. The method will read in the median results generated by the first method where the parameter value is its calibrated value. Then, it will, in turn, read in the median results for every other parameter value. So, for example, the minium value for chemoLowerLinearAdjust is 0.015. The method will read in the medians for all simulation runs for this value. For each output measure, the sets of median values are then compared using the Vargha-Delaney A-Test (2000), which indicates 'how different' the two sets of results are, or the effect the parameter value change has had on the simulation result. The next value for this parameter is then considered, and the same process followed. Once this is all complete, the A-Test scores for each parameter value comparison are output to a CSV file.

8. Return to the command prompt and run the R script using the same command as previously. This will then generate a results file for each parameter, within that parameters folder, summarising the A-Test results for all values it has been assigned. These files will take the name set in the R variable ATESTRESULTSFILENAME. Navigate to one of the parameter folders and make sure you are familiar with the structure of this file.

9. Go back to the text editor. We are now going to use the two methods within the package to represent the results generated previously in a graphical format. Add the following to the script text file:

```
oat_graphATestsForSampleSize(FILEPATH,PARAMETERS,PMIN,
PMAX,PINC,MEASURES,ATESTSIGLEVEL,
ATESTRESULTSFILENAME,TIMEPOINTS,TIMEPOINTSCALE)

oat_plotResultDistribution(FILEPATH,PARAMETERS,PMIN,
PMAX,PINC,MEASURES,MEASURE_SCALE,MEDIANSFILEFORMAT,
MEDIANSFILENAME,TIMEPOINTS,TIMEPOINTSCALE)
```

10. Return to the command prompt and run the R script using the same command as previously.

The first of these methods produces a graph for each parameter, which shows the A-Test scores for each value it has been assigned, when compared to its calibrated value. This plot will show all simulation output measures on the same graph. An example of the graphs that should have been produced can be seen in Figures 2 and 3. This helps identify any trends that become apparent when the value of a parameter is changed. For example, a notable change in simulation behaviour is identified when the value of chemoLowerLinearAdjust is perturbed (Figure 2). Or on the other hand, it may become apparent that the value of the parameter can be set to any value in the range and there be no notable change in simulation behaviour. This is the case with the chemoUpperLinearAdjust parameter, in Figure 3.

The final method again produces a graph for each parameter, but this time also for each simulation output measure. This generates a box plot showing the distribution of simulation results for that measure, for each parameter value, again making it easy to identify any trends in simulation behaviour against parameter value. When run, your script should have produced the graph seen in Figure 4.

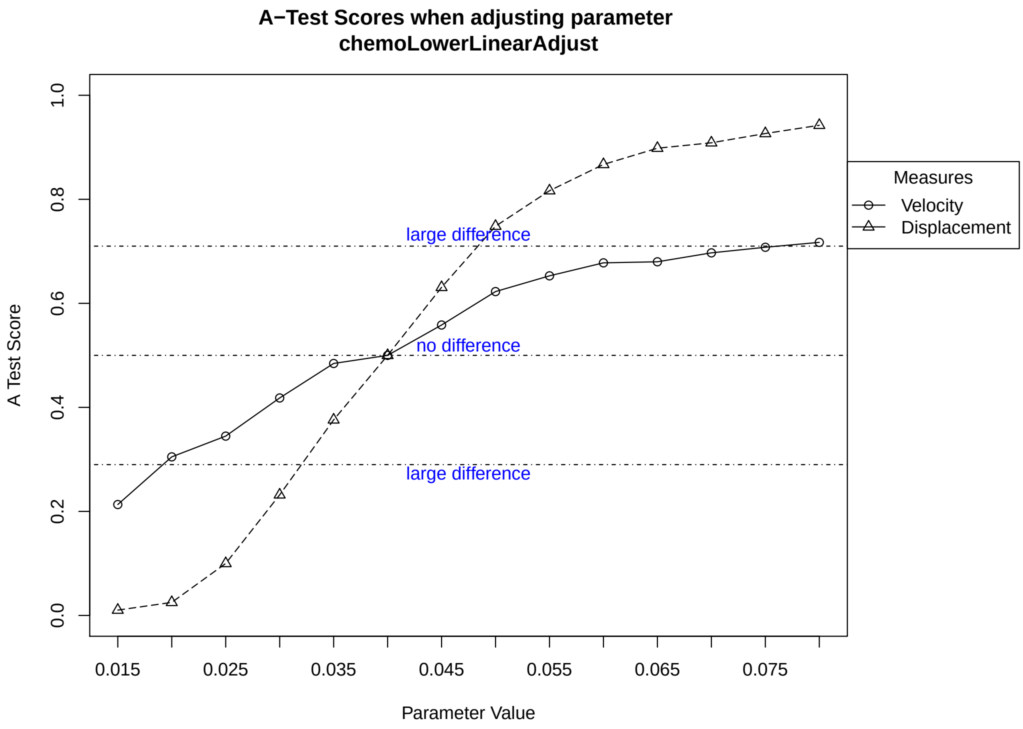

Figure 2: Graph showing the A-Test scores for all parameter values assigned to chemoLowerLinearAdjust

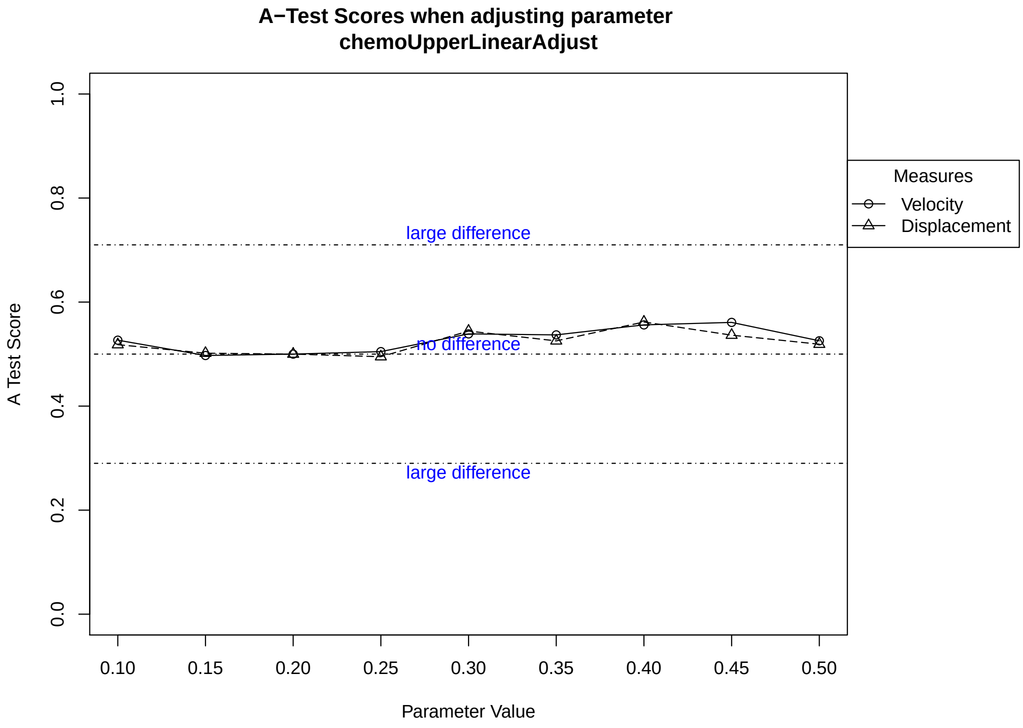

Figure 3: Graph showing the A-Test scores for all parameter values assigned to chemoUpperLinearAdjust

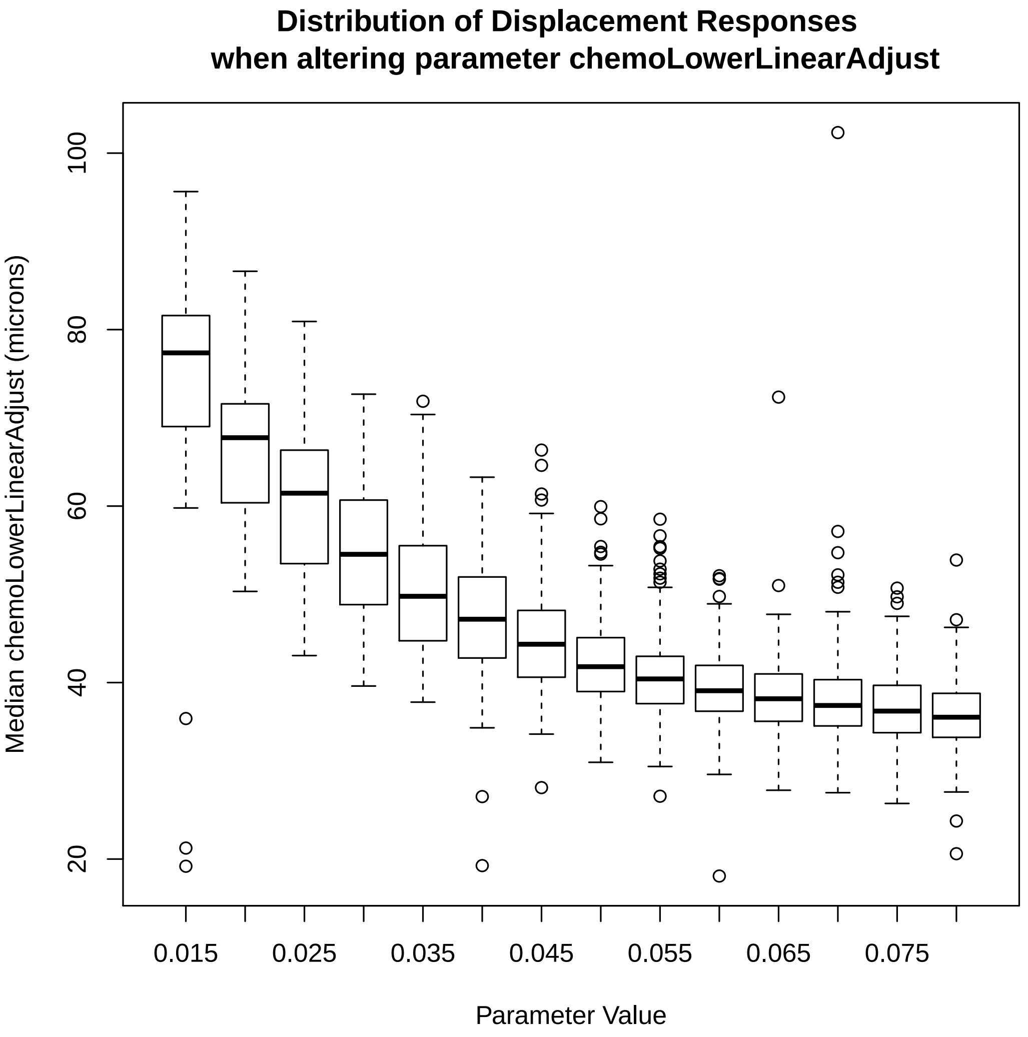

Figure 4: Graph showing the distribution of Displacement results when parameter chemoLowerLinearAdjust is perturbed

9 Running OAT Technique for Multiple Timepoints

The package also has the capability to perform the above analysis for simulation results taken at different timepoints. This may give an indication of when a parameter perturbation becomes influential. Again, we will examine this with an example, yet there is not much to change from the example seen previously

In this case study, we have captured the cell behaviour measures at multiple timepoints in the simulation, specifically 12, 36, 48,and 60 hours. Thus we have the output files trackedCells_Close_12.csv, trackedCells_Close_36.csv etc. To use this method over multiple timepoints, you should have (a) the same folder structure as in the previous example, and (b) an output file for each timepoint, with the timepoint appended to the filename after an underscore. It is worth writing a script to put your output in this format before looking at this method if this is not already the case.

We explain how this works through an example, which adapts what we did previously. Results at the 12, 36, 48, and 60 hour timepoints are contained within the example dataset used in the previous example.

1. Open the R script that you generated above in a text editor.

2. We now need to change the value of some of the variables. These are:

   ```
   TIMEPOINTS, TIMEPOINTSCALE
   ```

   Change the values so they match the below:

   ```
   TIMEPOINTS<-c(12,36,48,60)
   TIMEPOINTSCALE<-"Hours"
   ```

   This alteration sets TIMEPOINTS to an array of timepoints being analysed, and TIMEPOINTSCALE to a string stating what these timepoints represent. The latter is used for graphing results in the final part of the method.

   Each R method will now process each timepoint in turn and append the timepoint being analysed onto the input and output file names, so that distinct results are output for each. For example, when the 12 hour timepoint is being examined, the file addresses will become trackedCells_Close_12.csv, EgSet_Medians_12.csv, etc.

3. In the text editor, delete the four R methods used previously (under the variable declarations) and add these methods in their place:

   ```
   oat_processParamSubsets_overTime(FILEPATH,PARAMETERS,
   PMIN,PMAX,PINC,NUMRUNSPERSAMPLE,MEASURES,
   RESULTFILEFORMAT,RESULTFILENAME,ALTERNATIVEFILENAME,
   OUTPUTCOLSTART,OUTPUTCOLEND,MEDIANSFILEFORMAT,
   MEDIANSFILENAME,TIMEPOINTS)

   oat_analyseAllParams_overTime(FILEPATH,PARAMETERS,BASELINE,
   PMIN,PMAX,PINC,MEASURES,MEDIANSFILEFORMAT,
   MEDIANSFILENAME,ATESTRESULTSFILENAME,TIMEPOINTS)

   oat_graphATestsForSampleSize_overTime(FILEPATH,PARAMETERS,
   PMIN,PMAX,PINC,ATESTRESULTSFILENAME,MEASURES,
   ATESTSIGLEVEL,TIMEPOINTS,TIMEPOINTSCALE)

   oat_plotResultDistribution_overTime(FILEPATH,PARAMETERS,
   PMIN,PMAX,PINC,MEDIANSFILEFORMAT,MEDIANSFILENAME,
   MEASURES,MEASURE_SCALE,TIMEPOINTS,TIMEPOINTSCALE)
   ```

   The subtle change you will notice is that TIMEPOINTS and TIMEPOINTSCALE are now added to the top two methods. When each method is called, the method goes through each timepoint in turn. It will prepare the input and output filenames as stated above (adding the timepoint), then uses the same method as used in our first example (where only the end time point was analysed). Thus, whereas the first example produced output for one timepoint, R will now generate the same but for a number of different timepoints. To note the timepoint that was analysed, the graphs will have the timepoint appended to the filename in the same way as described previously.

4. Save the text file and run the script in R using the same command as previously

10 Further Reading

The following references may be useful in understanding this technique in more detail:

- Read, M., Andrews, P.S., Timmis, J. & Kumar, V. (2012) Techniques for Grounding Agent-Based Simulations in the Real Domain : a case study in Experimental Autoimmune Encephalomyelitis. Mathematical and Computer Modelling of Dynamical Systems, 18(1):67-86.

- Vargha, A. & Delaney, H.D. (2000) A critique and improvement of the CL Common Language Effect Size Statistics of McGraw and Wong. Journal of Educational and Behavioural Statistics, 25, p.pp.101-132.

- The spartan Manual, spartan-Manual.pdf, within the spartan package describes in more detail each method within the package
