# Supplementary material for: Spartan: A Comprehensive Tool for Understanding Uncertainty in Simulations of Biological Systems
Source: PLoS Comput Biol. 2013 Feb 28;9(2):e1002916. doi: 10.1371/journal.pcbi.1002916 (PMC3585389; doi:10.1371/journal.pcbi.1002916)
Supplement: Software S1 — Spartan R package for Linux and Mac OS. Includes tutorials for each technique. (ZIP) [file pcbi.1002916.s001.zip › Technique1_Tutorial.pdf]

Tutorial on using the *spartan* package to analyse agent-based simulation results

For use with *spartan* package version 1.2 onwards

Technique 1: Analysing Aleatory Uncertainty

1 Introduction

spartan, or (**S**imulation **P**arameter **A**nalysis **R** **T**oolkit **A**pplicatio**N**) is an R package which aids the understanding of the effect aleatory and epistemic uncertainty have on the output from a simulation. This set of tutorials makes use of available example simulation output to demonstrate how a variety of methods can be applied to further understand the results that have been generated. Following through each example should make it easier to apply the tookit to results generated by any agent-based computer simulation. This tutorial focuses on understanding the effect inherent stochasticity may have on simulation results.

2 The *spartan* Package

Computer simulations are becoming a popular technique to use in attempts to further our understanding of complex systems. This package provides code for four techniques described in available literature which aid the analysis of simulation results, at both single and multiple timepoints in the simulation run. The first technique addresses aleatory uncertainty in the system caused through inherent stochasticity, and determines the number of replicate runs necessary to generate a representative result. The second examines how robust a simulation is to parameter perturbation, through the use of a one-at-a-time parameter analysis technique. Thirdly, a latin hypercube based sensitivity analysis technique is included which can elucidate non-linear effects between parameters and indicate implications of epistemic uncertainty with reference to the system being modelled. Finally, a further sensitivity analysis technique, the extended Fourier Amplitude Sampling Test (eFAST) has been included to partition the variance in simulation results between input parameters, to determine the parameters which have a significant effect on simulation behaviour.

3 The Case Study

The example simulation results have been taken from an ongoing project which seeks to understand the formation of lymphoid tissue in the small intestine. This simulation outputs cell behaviour measures at various points in the simulation and measures describing the development of the tissue, which occurs through interactions between the cells. Techniques 2-4 of this package allow us to explore how input parameter value affects the behaviour of these cells. We need Technique 1 to tell us how many simulation runs we need for each condition explored to ensure we have a robust representative result.

4 Scope

Do note that the idea of this tutorial is to demonstrate the application of the toolkit, and is not intended to act as a full introduction to using Sensitivity Analysis techniques in the analysis of simulation results. Where useful, links to further reading have been included.

5 Prerequisites

- The R statistical environment, version 2.13.1 or later.
- The spartan R package, downloaded from the Comprehensive R Archive Network (CRAN) or from the project website.
- The lhs and gplots R packages, available for download from CRAN.
- The example simulation results, available from the project website.
- From version 1.2 of *spartan*, simulation results can be in either CSV or XML format. For earlier versions, results must be pre-processed to be in CSV format.

6 Running Technique 1: Aleatory Analysis (AA)

Aleatory uncertainty can be caused by inherent stochasticity within a simulation. This is especially the case for agent-based simulations, where different results will be gained although the same parameter input values used. Thus a number of replicate simulation runs need to be performed to achieve a representative result. This technique gives an indication of the number of simulation runs necessary to reduce this uncertainty. This follows the method described by Read et al (2012). Note at this stage we are not concerned with parameter sensitivity, more producing a representative result. Thus this analysis uses the simulation parameters set through some process of calibration.

To use this, you should have chosen the number of replicate runs to analyse. For example, in this tutorial we examine 1, 5, 50, 100 and 300 replicates of a simulation under the same parameter conditions. For each sample, twenty sets of results are created, with each set containing the number of simulation results for that sample size. So, we have 20 sets where the simulation was run once, 20 sets where each set contains the results of 5 runs, right through to 20 sets where each contains the results of 300 runs. The technique then looks at each sample size used, and (a) generates the median distribution for all output measures for each of the twenty subsets (b) goes through each of the subsets, comparing the median distribution of each measure with the respective median distribution in the first subset using the Vargha-Delaney A-Test (2000) which gives an indication of how different the results are, (c) for each sample size, creates a graph showing how different the results of each subset are (i.e. the A-Test result).

This section of the tutorial performs this analysis for the lymphoid tissue formation simulation, which has been developed using an agent-based approach. In this case, we are going to examine two cell behaviour measures, Velocity and Displacement, that are captured for a simulation period representing one hour. In the simulation results folders, the file we are going to look at is trackedCells_Close_Endpoint.csv.

1. Download the AA example result set from the project website and extract the results.
2. The first thing to note is the folder structure. To use this method, the simulation results do need to be in a specific format (Figure 1 AA folder structure). The structure has three levels:
   (i) Folders for each sample size being analysed
   (ii) Twenty folders, one for each of the subsets within that sample size
   (iii) A folder for each of the simulation results in that subset. So, if you are analysing five simulation results, there will be 5 folders, numbered 1-5.

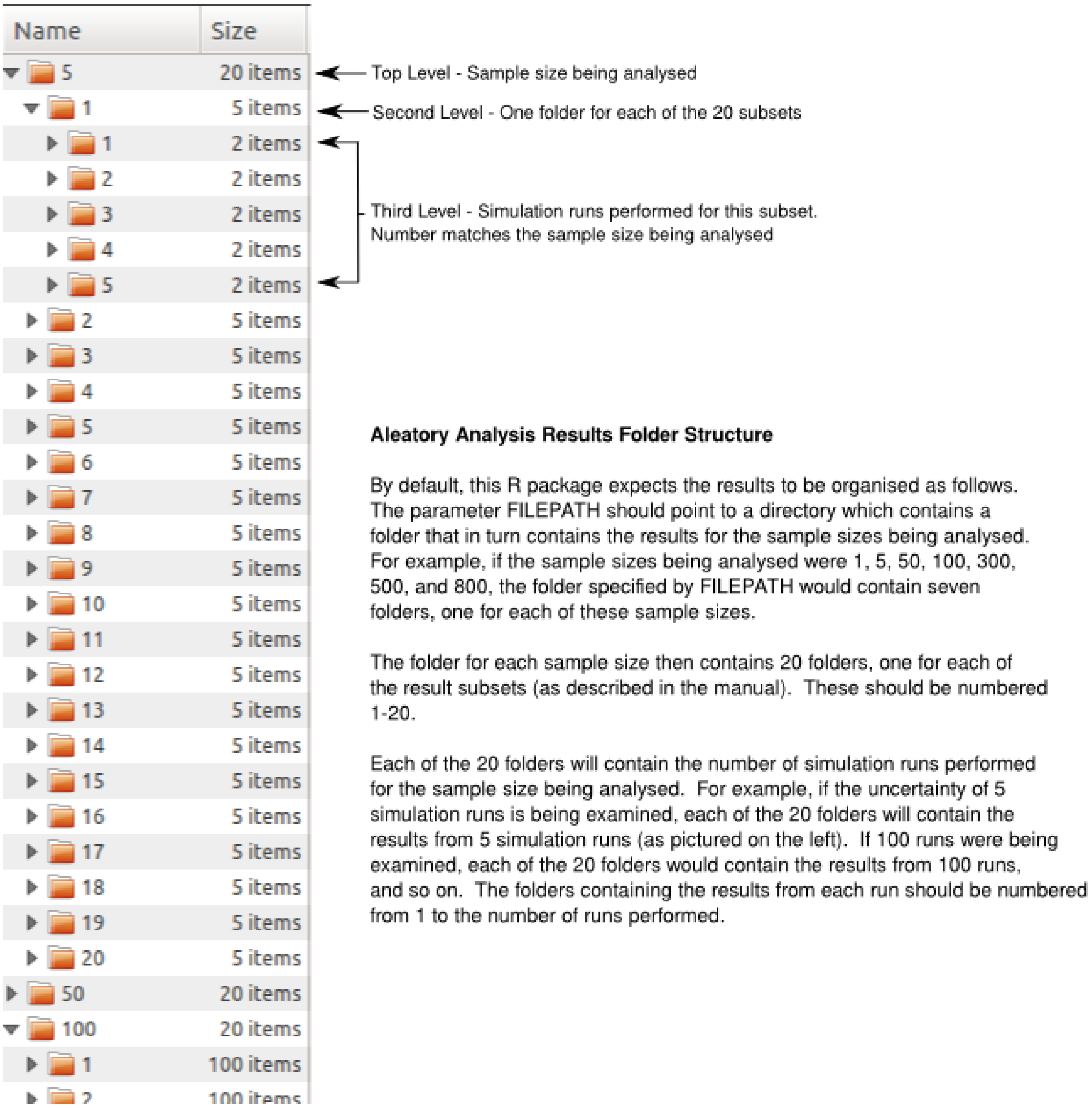

Figure 1: Simulation results folder structure that should exist for use with this tool

3. With this data available, open a text editor (gedit or similar). Now we are going to declare the R variables required to run this analysis. Type or copy in the text on Page 4. Firstly, the *spartan* library is imported. The R variables required for this analysis are then declared in capital letters. The line underneath the variable, beginning with a #, is a description of that being declared. Make sure you set the FILEPATH variable to where you have extracted the tutorial data.

```
library(spartan)
# Import the package

FILEPATH <- "/media/FreeAgent/package_Test_Data/AA"
# The directory where you have extracted the example simulation results.
SAMPLESIZES <- c(1,5,50,100,300)
# The sample sizes that are to be analysed, contained within an array
MEASURES<-c("Velocity","Displacement")
# The simulation output measures to be analysed, again contained within an array
NUMSUBSETSPERSAMPLESIZE<-20
# The number of subsets used.  By default use 20, as performed by Read et al in
# their published technique
RESULTFILEFORMAT<-"csv"
# Simulation result file format. Should be either CSV or XML
RESULTFILENAME<-"trackedCells_Close"
# The output file containing the simulation results from that simulation run. Note
# there should be no file extension
ALTFILENAME<-NULL
# Not used in this case, but this is useful in cases where two result files may
# exist (for example if tracking cells close to an area, and those further away
#  two output files could be used).  Here, results in a second file are processed
# if the first is blank or does not exist.
OUTPUTFILECOLSTART<-10
# Use this if simulation results are in CSV format.
# The column within the csv results file where the results start.  This is useful
# as it restricts what is read in to R, getting round potential errors where the
# first column contains an agent label (as R does not read in CSV files where the
# first column contains duplicates)
OUTPUTFILECOLEND<-11
# Use this if simulation results are in CSV format.
# Last column of the output measure results
MEDIANSFILEFORMAT<-"csv"
# File format of median results file that will be generated in this tutorial.
# Should be either XML or CSV. This is used as, for some applications, a simulation
# results set for processing may have been generated using methods other than
# spartan, and may not be in CSV format.
MEDIANSFILENAME<-"EgSet_Medians"
# For each of the twenty subsets, a file is created containing the median of each
# output measure, of each simulation run in that subset.  This parameter sets the
# name of this file. Note no file extension
ATESTRESULTFILENAME<-"EgSet_ATests"
# The results of the A-Test comparisons of the twenty subsets for each sample size
# are stored within an output file.  This parameter sets the name of this file.
# Note no file extension. Current versions of spartan output to CSV files, though
# this may change in later versions.
SUMMARYFILENAME<-"EgSet_ATestMaxAndMedians"
```

```
# In this example, we will have created an EgSet_ATests.csv file for sample sizes
# 1,5,50,100 and 300. A summary file is created containing the maximum and median
# A-Test values for each sample size. This parameter sets the name of this file.
# Again note no file extension
LARGEDIFFINDICATOR<-0.23
# The A-Test value either side of 0.5 which should be considered a 'large difference'
# between two sets of results.  Use of 0.23 was taken from the Vargha-Delaney
# publication but can be adjusted here as necessary.
SMALL<-0.56
MEDIUM<-0.66
LARGE<-0.73
# A-Test values above 0.5 (no difference) which should be considered as small,
# medium, and large differences between two result sets.  Used in the graph
# summarising all sample sizes.
GRAPHOUTPUTFILE<-"EgSet_ATestMaxes"
# Name of the graph which summarises the analysis results for all sample sizes.
# Current versions of spartan output to pdf. Note no file extension
TIMEPOINTS<-NULL; TIMEPOINTSCALE<-NULL
# Not used in this case, but when a simulation is analysed at multiple timepoints
# (see later in tutorial)
```

4. Now to run the first of the three methods (we are going to do each individually in the tutorial so the functionality becomes apparent but in reality you will run all three methods one after another in the same text file). Copy the below into the text file under the above declarations:

   ```
   aa_analyse_all_sample_sizes(FILEPATH, SAMPLESIZES, NUMSUBSETSPERSAMPLESIZE,
   RESULTFILEFORMAT, RESULTFILENAME, ALTFILENAME, OUTPUTFILECOLSTART,
   OUTPUTFILECOLEND, MEASURES, MEDIANSFILEFORMAT,MEDIANSFILENAME,
   ATESTRESULTFILENAME, LARGEDIFFINDICATOR)
   ```

   This independently examines each sample size to be examined, and determines how 'different' the results of each of the 20 subsets are. As a first step, the algorithm needs to go through each subset and create a set of medians which can then be compared with another of the 20 subsets. Therefore, the algorithm takes each of the 20 sets in turn, producing a file containing the median of each output measure for each simulation run in that set. This may be easier to understand with the use of an example. Lets say we are analysing the uncertainty in 5 simulation runs. So our sample size is 5. For this sample size, we will have 20 sets of results, each containing 5 runs. The algorithm takes each of these 20 in turn, producing a file containing the median output for all measures for each of the 5 runs. (so, in our example, we have two output measures, Velocity and Displacement - a file would be created within each of the 20 sets containing the median Velocity and Displacement measures for each of the 5 runs). Once this process has been completed, the median results for subsets 2-20 are compared with those in subset 1 using the Vargha-Delaney A-Test, with these results stored in an output file. This file is either in CSV or XML format, dependent on the file format set in the MEDIANSFILEFORMAT variable. The A-Test results are then graphed, showing how different each of the 20 subsets are. Thus, when this example is run, you will generate five graphs, one for each sample size. The graph you should generate for a sample size of 5 (AA_5Samples.pdf) is in Figure 2.

5. Save the file with a suitable filename and .R extension (for example AA_Analysis.R)

6. For Linux or Mac Operating Systems, open a command prompt, and navigate to the directory where this file was saved. Type the following:

```
Rscript AA_Analysis.R
```

If you are using Windows, open R and type the following into the R Command Prompt (where [*path to directory* is the full path to where AA_Analysis.R is saved]:

```
source("C:\[\textit{path to directory}]\AA_Analysis.R")
```

For the set of parameters specified above, this will produce:

(i) Median results files within each of the 20 folders within each sample size - named as stated in the MEDIANSFILENAME variable (EgSet_Median.csv in this case)

(ii) A graph within each sample size, showing 'how different' result sets 2-20 are in comparison with the results in subset 1. These graphs should be named *n*Samples.pdf.

7. We are now going to run the second method, which produces a summary of the results for all sample sizes. Go back to your R script in the text editor, and add the following to the file:

```
aa_sampleSizeSummary(FILEPATH,SAMPLESIZES,MEASURES,ATESTRESULTFILENAME,
SUMMARYFILENAME)
```

Save the file, go back to the command prompt, and run the same R command as previously. This will produce, in the top level of the folder structure, a csv file named as specified by SUMMARYFILENAME (EgSet_ATestMaxAndMedians.csv in this case). If you open this CSV file, you will see it contains both the maximum and median A-Test scores over the 20 subsets for each output measure, and for each sample size. Alongside these values you will also find a 'Normalised' value. With the A-Test, a result of 0.5 implies there is no significant difference. The directionality of results either side of this is not important, and therefore these can be normalised so that all A-Test maximum and median values are above 0.5.

8. The final method creates a graph of the maximum A-Test score for each sample size. Back in the text editor, add the following to the R script file:

```
aa_graphSampleSizeSummary(FILEPATH,MEASURES, 300,SMALL, MEDIUM, LARGE,
SUMMARYFILENAME, GRAPHOUTPUTFILE, TIMEPOINTS,TIMEPOINTSCALE)
```

Note that the 300 is the maximum sample size analysed. Save the file and run the script in R. This will produce a summary graph in the top level of the folder structure containing the maximum A-Test value observed over the 20 subsets for each sample size. You should generate the same graph as that in Figure 3. All being well, you should see the score decrease as sample size increases.

Your aim is to find a sample size which minimises the A-Test value for all measures. In this example case, we did not deem 300 runs to be sufficiently close enough to the 'no difference' line for both Velocity and Displacement measures, and did the same analysis with 500 runs. However, this value will be different for all simulations that are analysed this way.

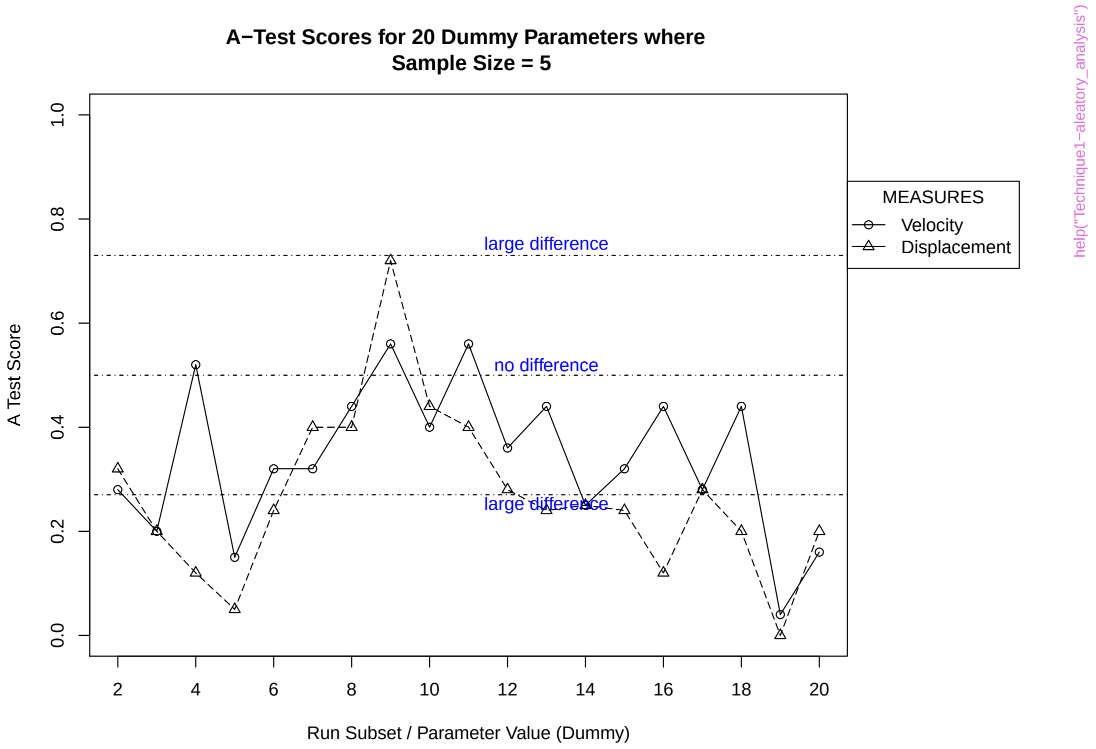

Figure 2: Graph showing the A-Test scores where sample sets 2-20 are compared with the first sample set

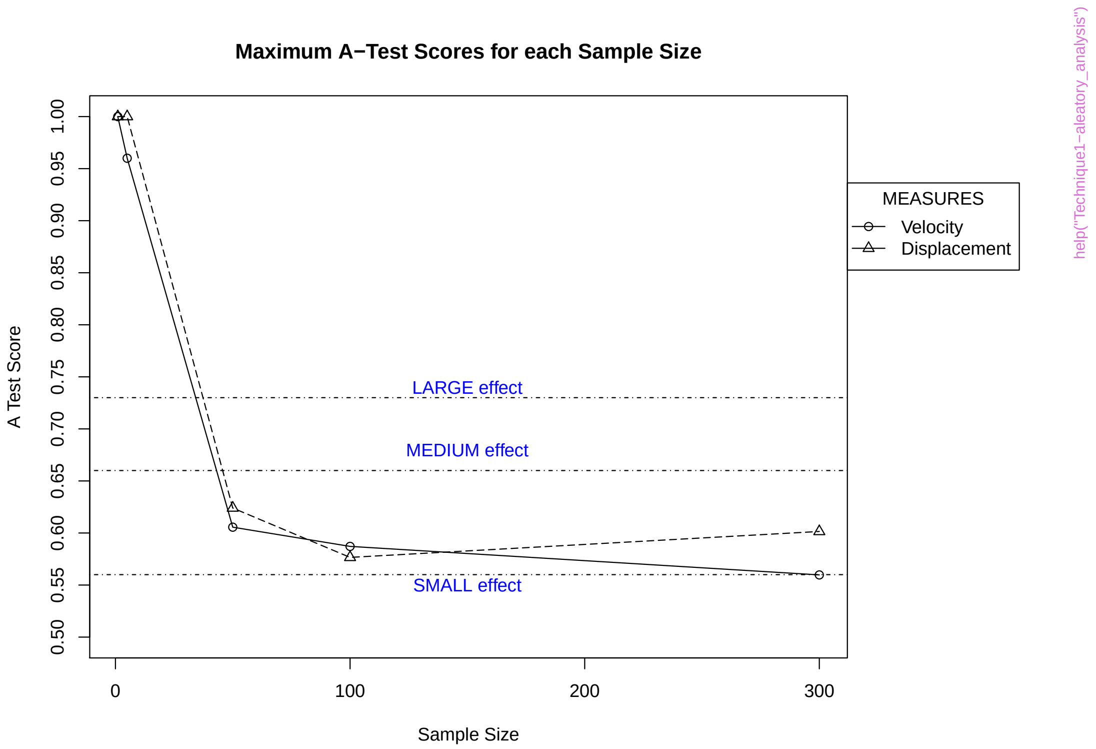

Figure 3: Graph summarising the maximum A-Test result observed over the 20 subsets, for all sample sizes

7 Running AA Technique for Multiple Timepoints

The package also has the capability to perform the above analysis for simulation results taken at different timepoints. This may give an indication of when such variance becomes an influential. Again, we will examine this with an example, yet there is not much to change from the example seen previously

In this case study, we have captured the cell behaviour measures at multiple timepoints in the simulation, specifically 12, 36, 48,and 60 hours. Thus we have the output files trackedCells_Close_12.csv, trackedCells_Close_36.csv etc. To use this method over multiple timepoints, you should have (a) the same folder structure as in the previous example, and (b) an output file for each timepoint, with the timepoint appended to the filename after an underscore. It is worth writing a script to put your output in this format before looking at this method if that is not already the case.

We explain how this works through an example, which adapts what we did previously. Results at the 12, 36, 48, and 60 hour timepoints are contained within the example dataset used in the previous example.

1. Open the R script that you generated above in a text editor.

2. We now need to alter the values of two variables. These are:

   ```
   TIMEPOINTS, TIMEPOINTSCALE
   ```

   Change the values so they match the below:

   ```
   TIMEPOINTS<-c(12,36,48,60)
   TIMEPOINTSCALE<-"Hours"
   ```

   This sets TIMEPOINTS to an array of timepoints being analysed, and TIMEPOINTSCALE to a string stating what these timepoints represent. The latter is used for graphing results in the final part of the method.

   Each R method will now process each timepoint in turn and append the timepoint being analysed onto the input and output file names, so that distinct results are output for each. For example, when the 12 hour timepoint is being examined, the file addresses will become trackedCells_Close_12.csv, EgSet_Medians_12.csv, etc.

3. In the text editor, delete the three R methods used previously (under the variable declarations) and add these three methods in their place:

   ```
   aa_analyse_all_sample_sizes_overTime(FILEPATH,SAMPLESIZES,
   NUMSUBSETSPERSAMPLESIZE,RESULTFILEFORMAT,RESULTFILENAME,
   ALTFILENAME,OUTPUTFILECOLSTART,OUTPUTFILECOLEND,MEASURES,
   MEDIANSFILEFORMAT,MEDIANSFILENAME,ATESTRESULTFILENAME,
   LARGEDIFFINDICATOR,TIMEPOINTS,TIMEPOINTSCALE)

   aa_sampleSizeSummary_overTime(FILEPATH,SAMPLESIZES,MEASURES,
   ATESTRESULTFILENAME,SUMMARYFILENAME,TIMEPOINTS)

   aa_graphSampleSizeSummary_overTime(FILEPATH,MEASURES,300,
   SMALL,MEDIUM,LARGE,SUMMARYFILENAME,TIMEPOINTS,
   TIMEPOINTSCALE)
   ```

The subtle change you will notice is that TIMEPOINTS and TIMEPOINTSCALE are now added to the top two methods. When each method is called, the method goes through each timepoint in turn. It will prepare the input and output filenames as stated above (adding the timepoint), then uses the same method as used in our first example (where only the end time point was analysed). Thus, whereas the first example produced graphs and a summary graph for one timepoint, R will now generate the same but for a number of different timepoints. To note the timepoint that was analysed, the graphs will have the timepoint appended to the filename in the same way as described previously.

4. Save the file in the text editor and run the script in R using the same command as previously.

8 Further Reading

The following two references may be useful in understanding this technique in more detail:

- Read, M., Andrews, P.S., Timmis, J. & Kumar, V. (2012) Techniques for Grounding Agent-Based Simulations in the Real Domain : a case study in Experimental Autoimmune Encephalomyelitis. Mathematical and Computer Modelling of Dynamical Systems, 18(1):67-86.
- Vargha, A. & Delaney, H.D. (2000) A critique and improvement of the CL Common Language Effect Size Statistics of McGraw and Wong. Journal of Educational and Behavioural Statistics, 25, p.pp.101-132.
- The spartan Manual, spartan-Manual.pdf, within the spartan package describes in more detail each method within the package
