# Supplementary material for: Spartan: A Comprehensive Tool for Understanding Uncertainty in Simulations of Biological Systems
Source: PLoS Comput Biol. 2013 Feb 28;9(2):e1002916. doi: 10.1371/journal.pcbi.1002916 (PMC3585389; doi:10.1371/journal.pcbi.1002916)
Supplement: Software S1 — Spartan R package for Linux and Mac OS. Includes tutorials for each technique. (ZIP) [file pcbi.1002916.s001.zip › Install_spartan.pdf]

Installing the *spartan* package for use in the R Statistical Environment

1 Introduction

spartan, or (**S**imulation **P**arameter **A**nalysis **R** **T**oolkit **A**pplicatio**N**) is an R package which aids the understanding of the effect aleatory and epistemic uncertainty have on the output from a simulation.

2 The *spartan* Package

Computer simulations are becoming a popular technique to use in attempts to further our understanding of complex systems. This package provides code for four techniques described in available literature which aid the analysis of simulation results, at both single and multiple timepoints in the simulation run. The first technique addresses aleatory uncertainty in the system caused through inherent stochasticity, and determines the number of replicate runs necessary to generate a representative result. The second examines how robust a simulation is to parameter perturbation, through the use of a one-at-a-time parameter analysis technique. Thirdly, a latin hypercube based sensitivity analysis technique is included which can elucidate non-linear effects between parameters and indicate implications of epistemic uncertainty with reference to the system being modelled. Finally, a further sensitivity analysis technique, the extended Fourier Amplitude Sampling Test (eFAST) has been included to partition the variance in simulation results between input parameters, to determine the parameters which have a significant effect on simulation behaviour.

3 Prerequisites

- The R statistical environment, version 2.13.1 or later.
- The spartan R package, downloaded from the Comprehensive R Archive Network (CRAN) or from the project website.
- The lhs and gplots R packages, available for download from CRAN.
- The example simulation results, available from the project website.
- From version 1.2 of *spartan*, simulation results can be in either CSV or XML format. For earlier versions, results must be pre-processed to be in CSV format.

4 Installing the Package

There are two ways to install the *spartan* package into your R environment:

1. **Linux/Mac: Download from the Website and Install From Source**

   Download the package from the project website. Open a terminal window and navigate to the directory where the spartan.tar.gz file has been saved. To install in the R default directory, type the following:

```
R CMD INSTALL spartan_1.2.tar.gz
```

To install to a specific directory, type the following:

```
R CMD INSTALL spartan_1.2.tar.gz -l /path/to/directory/
```

2. **Install Directly from CRAN**

   Open the R environment (Linux/Mac: type R in most cases, Windows: Open from Programs menu). Enter the following at the prompt:

   ```
   install.packages("spartan")
   ```

   Or, to install to a specific directory, type the following:

   ```
   install.packages("spartan",lib="/path/to/directory")
   ```

5 Loading the Package

To use the functionality of the package, declare the following at the top of your script

```
library(spartan)
```

Or, if you have installed the package in another directory than R's default package store:

```
library(spartan,lib.loc="/path/to/directory")
```
