# Supplementary material for: Spartan: A Comprehensive Tool for Understanding Uncertainty in Simulations of Biological Systems
Source: PLoS Comput Biol. 2013 Feb 28;9(2):e1002916. doi: 10.1371/journal.pcbi.1002916 (PMC3585389; doi:10.1371/journal.pcbi.1002916)
Supplement: Software S1 — Spartan R package for Linux and Mac OS. Includes tutorials for each technique. (ZIP) [file pcbi.1002916.s001.zip › spartan_1.2.tar.gz!/spartan/inst/extdata/eFAST_Velocity.pdf]

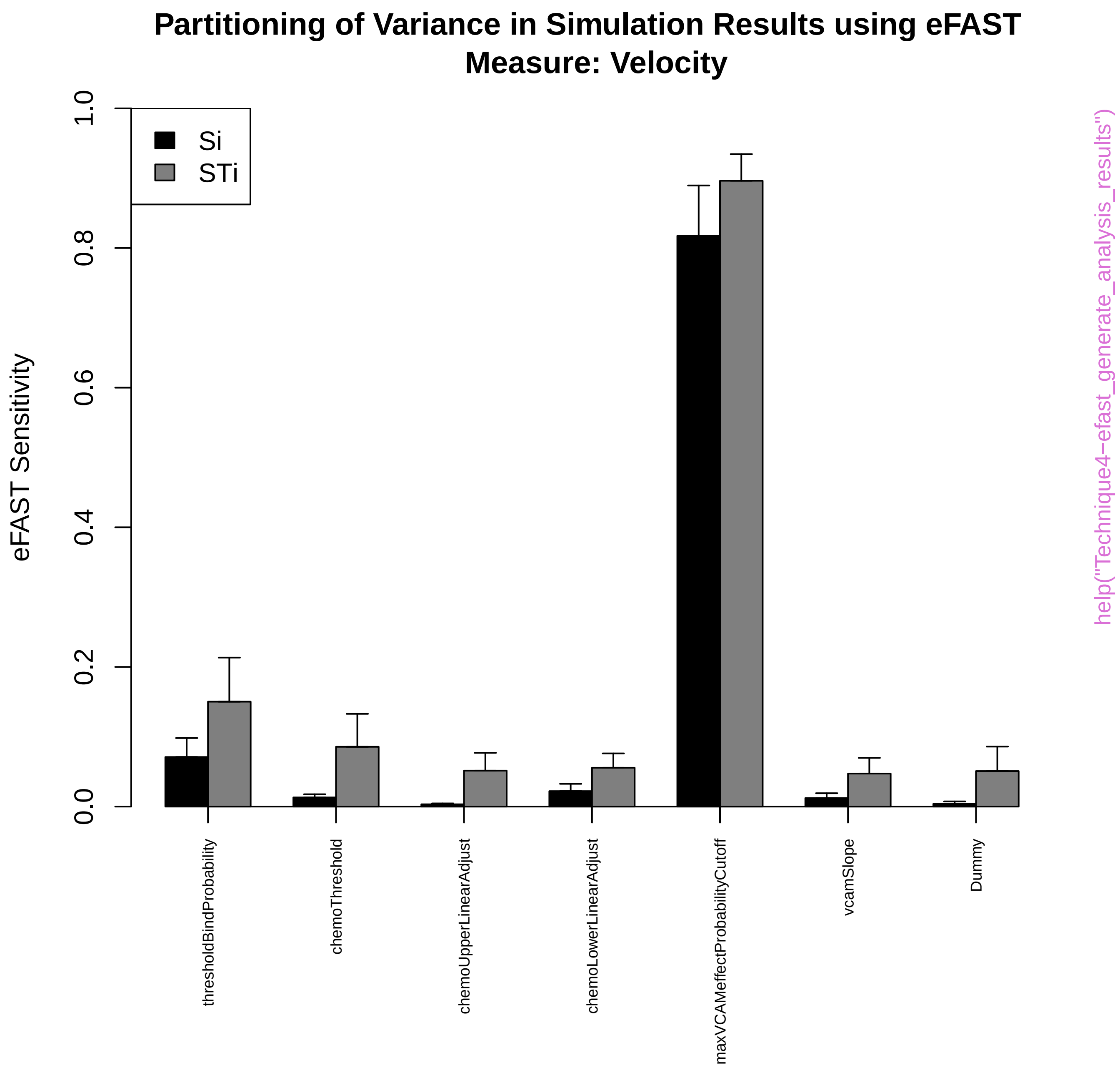

Partitioning of Variance in Simulation Results using eFAST
Measure: Velocity
eFAST Sensitivity
1.0
0.8
0.6
0.4
0.2
0.0
Si
STi
thresholdBindProbability
chemoThreshold
chemoUpperLinearAdjust
chemoLowerLinearAdjust
maxVCAMeffectProbabilityCutoff
vcamSlope
Dummy
help("Technique4-efast_generate_analysis_results")
