# Supplementary material for: Spartan: A Comprehensive Tool for Understanding Uncertainty in Simulations of Biological Systems
Source: PLoS Comput Biol. 2013 Feb 28;9(2):e1002916. doi: 10.1371/journal.pcbi.1002916 (PMC3585389; doi:10.1371/journal.pcbi.1002916)
Supplement: Software S1 — Spartan R package for Linux and Mac OS. Includes tutorials for each technique. (ZIP) [file pcbi.1002916.s001.zip › spartan_1.2.tar.gz!/spartan/inst/extdata/chemoLowerLinearAdjust_DisplacementBP.pdf]

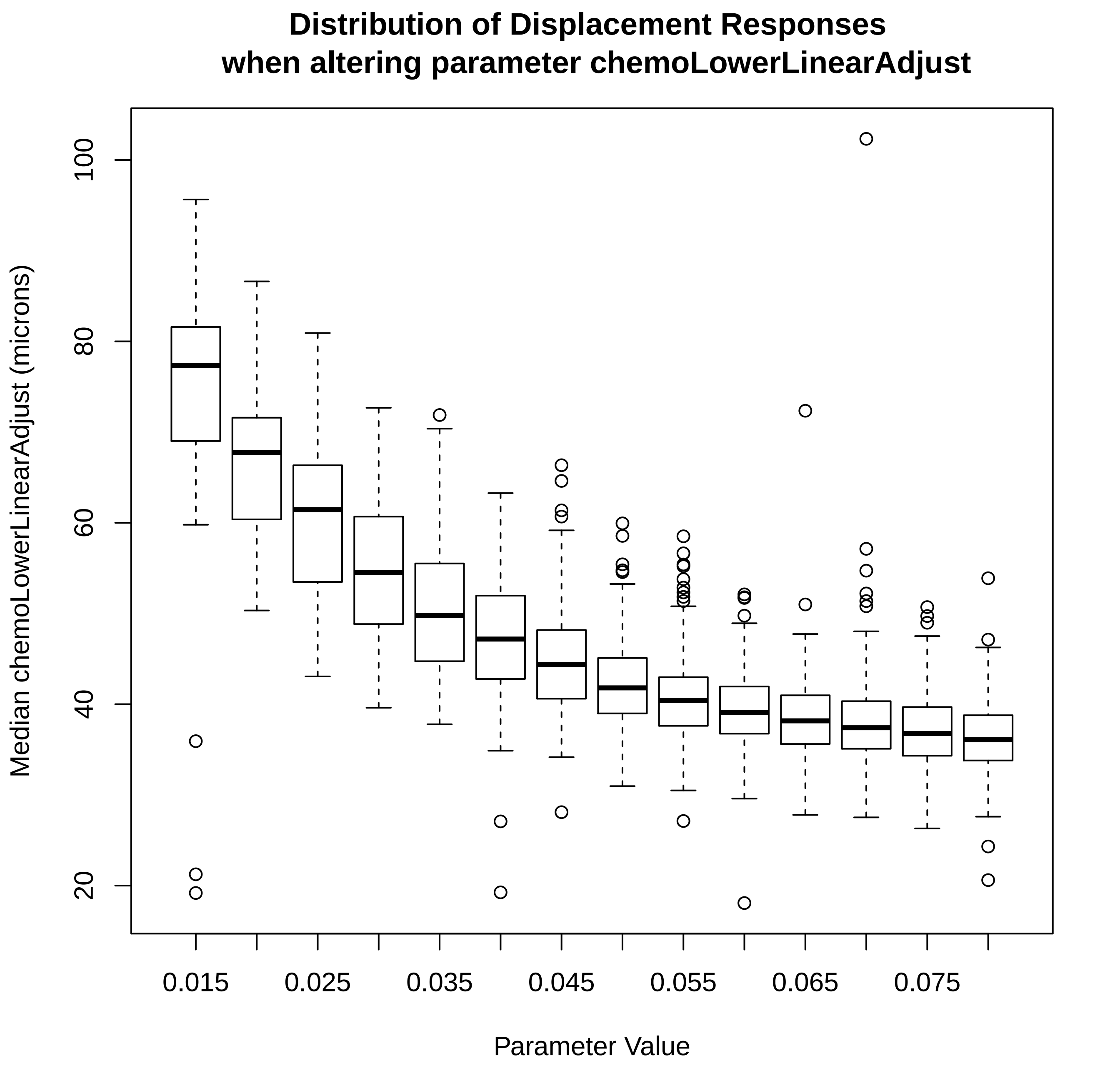

Distribution of Displacement Responses
when altering parameter chemoLowerLinearAdjust
Median chemoLowerLinearAdjust (microns)
100
80
60
40
20
0.015
0.025
0.035
0.045
0.055
0.065
0.075
Parameter Value
