# Supplementary material for: Spartan: A Comprehensive Tool for Understanding Uncertainty in Simulations of Biological Systems
Source: PLoS Comput Biol. 2013 Feb 28;9(2):e1002916. doi: 10.1371/journal.pcbi.1002916 (PMC3585389; doi:10.1371/journal.pcbi.1002916)
Supplement: Software S1 — Spartan R package for Linux and Mac OS. Includes tutorials for each technique. (ZIP) [file pcbi.1002916.s001.zip › spartan_1.2.tar.gz!/spartan/inst/extdata/AA_Results.pdf]

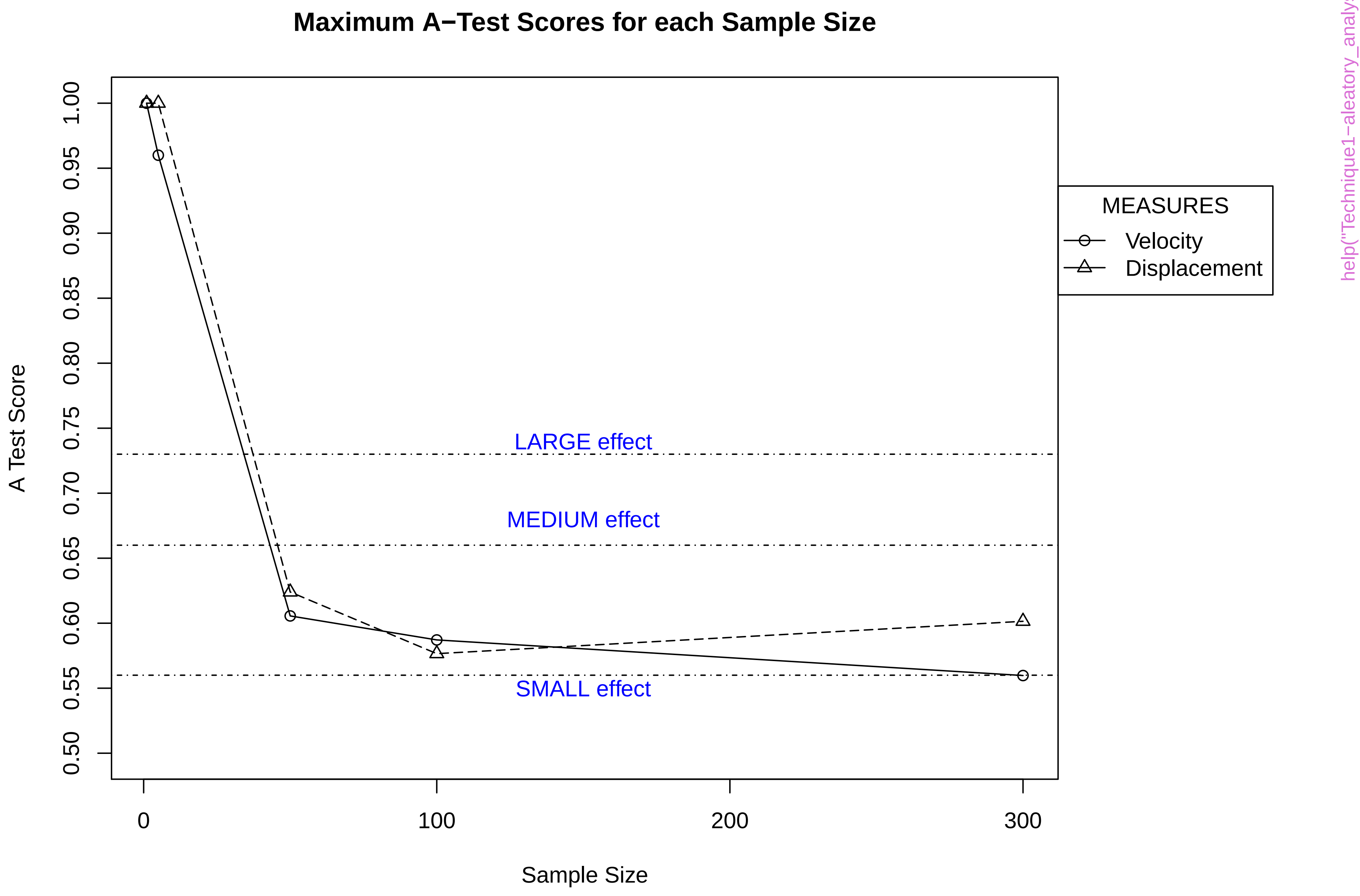
Maximum A−Test Scores for each Sample Size
A Test Score
1.00
0.95
0.90
0.85
0.80
0.75
0.70
0.65
0.60
0.55
0.50
LARGE effect
MEDIUM effect
SMALL effect
0
100
200
300
Sample Size
MEASURES
Velocity
Displacement
help("Technique1−aleatory_analysis")
