# Supplementary material for: Spartan: A Comprehensive Tool for Understanding Uncertainty in Simulations of Biological Systems
Source: PLoS Comput Biol. 2013 Feb 28;9(2):e1002916. doi: 10.1371/journal.pcbi.1002916 (PMC3585389; doi:10.1371/journal.pcbi.1002916)
Supplement: Software S1 — Spartan R package for Linux and Mac OS. Includes tutorials for each technique. (ZIP) [file pcbi.1002916.s001.zip › spartan_1.2.tar.gz!/spartan/inst/extdata/OAT_chemoUpperLinearAdjust.pdf]

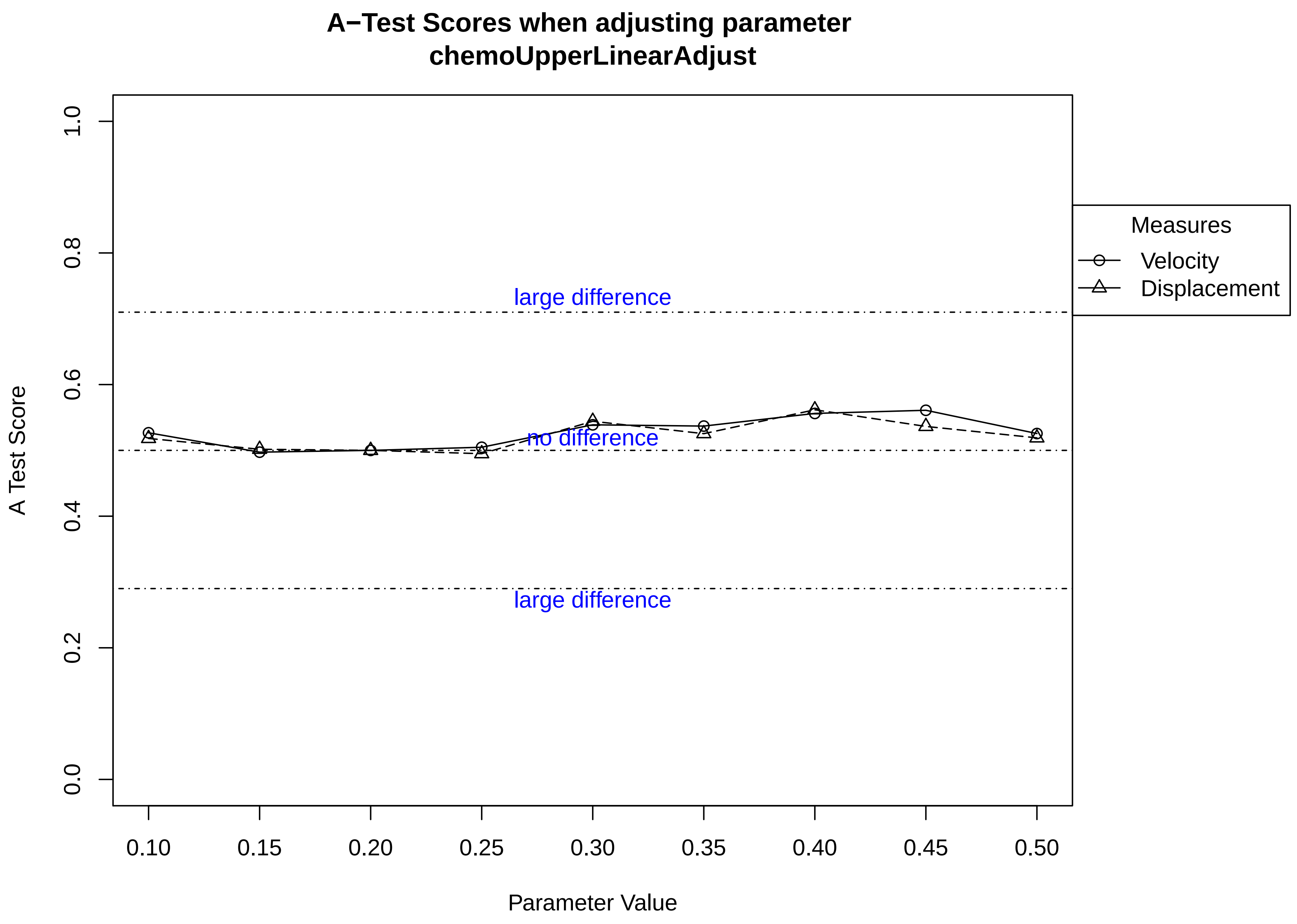
A−Test Scores when adjusting parameter
chemoUpperLinearAdjust
A Test Score
0.0
0.2
0.4
0.6
0.8
1.0
large difference
no difference
large difference
0.10
0.15
0.20
0.25
0.30
0.35
0.40
0.45
0.50
Parameter Value
Measures
Velocity
Displacement
