# Supplementary material for: Spartan: A Comprehensive Tool for Understanding Uncertainty in Simulations of Biological Systems
Source: PLoS Comput Biol. 2013 Feb 28;9(2):e1002916. doi: 10.1371/journal.pcbi.1002916 (PMC3585389; doi:10.1371/journal.pcbi.1002916)
Supplement: Software S1 — Spartan R package for Linux and Mac OS. Includes tutorials for each technique. (ZIP) [file pcbi.1002916.s001.zip › spartan_1.2.tar.gz!/spartan/inst/extdata/LHC_maxVCAMeffectProbabilityCutoff_Velocity.pdf]

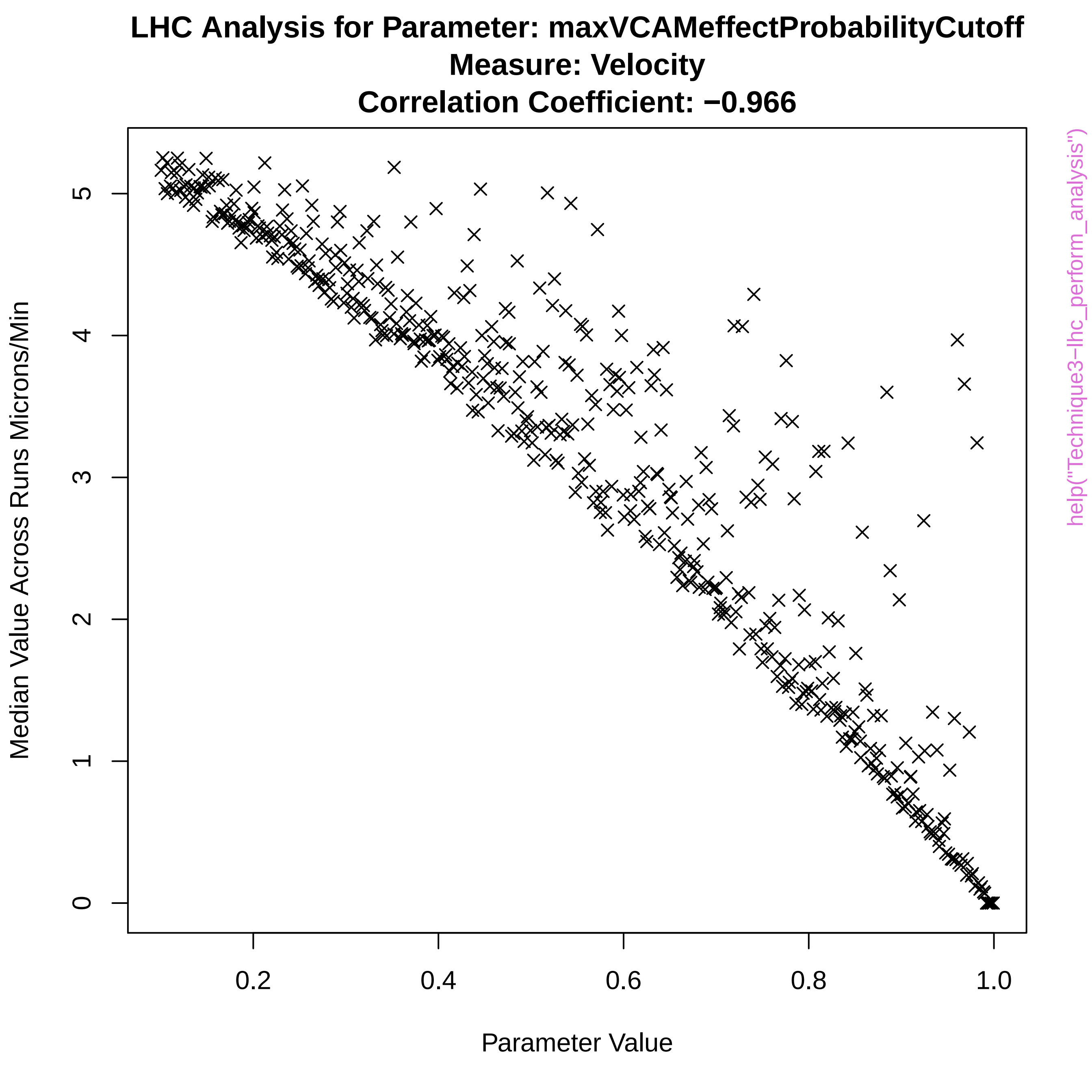
LHC Analysis for Parameter: maxVCAMeffectProbabilityCutoff
Measure: Velocity
Correlation Coefficient: −0.966
Median Value Across Runs Microns/Min
Parameter Value
0
1
2
3
4
5
0.2
0.4
0.6
0.8
1.0
help("Technique3−lhc_perform_analysis")
