# Supplementary material for: Spartan: A Comprehensive Tool for Understanding Uncertainty in Simulations of Biological Systems
Source: PLoS Comput Biol. 2013 Feb 28;9(2):e1002916. doi: 10.1371/journal.pcbi.1002916 (PMC3585389; doi:10.1371/journal.pcbi.1002916)
Supplement: Software S1 — Spartan R package for Linux and Mac OS. Includes tutorials for each technique. (ZIP) [file pcbi.1002916.s001.zip › spartan_1.2.tar.gz!/spartan/inst/extdata/AA_5Samples.pdf]

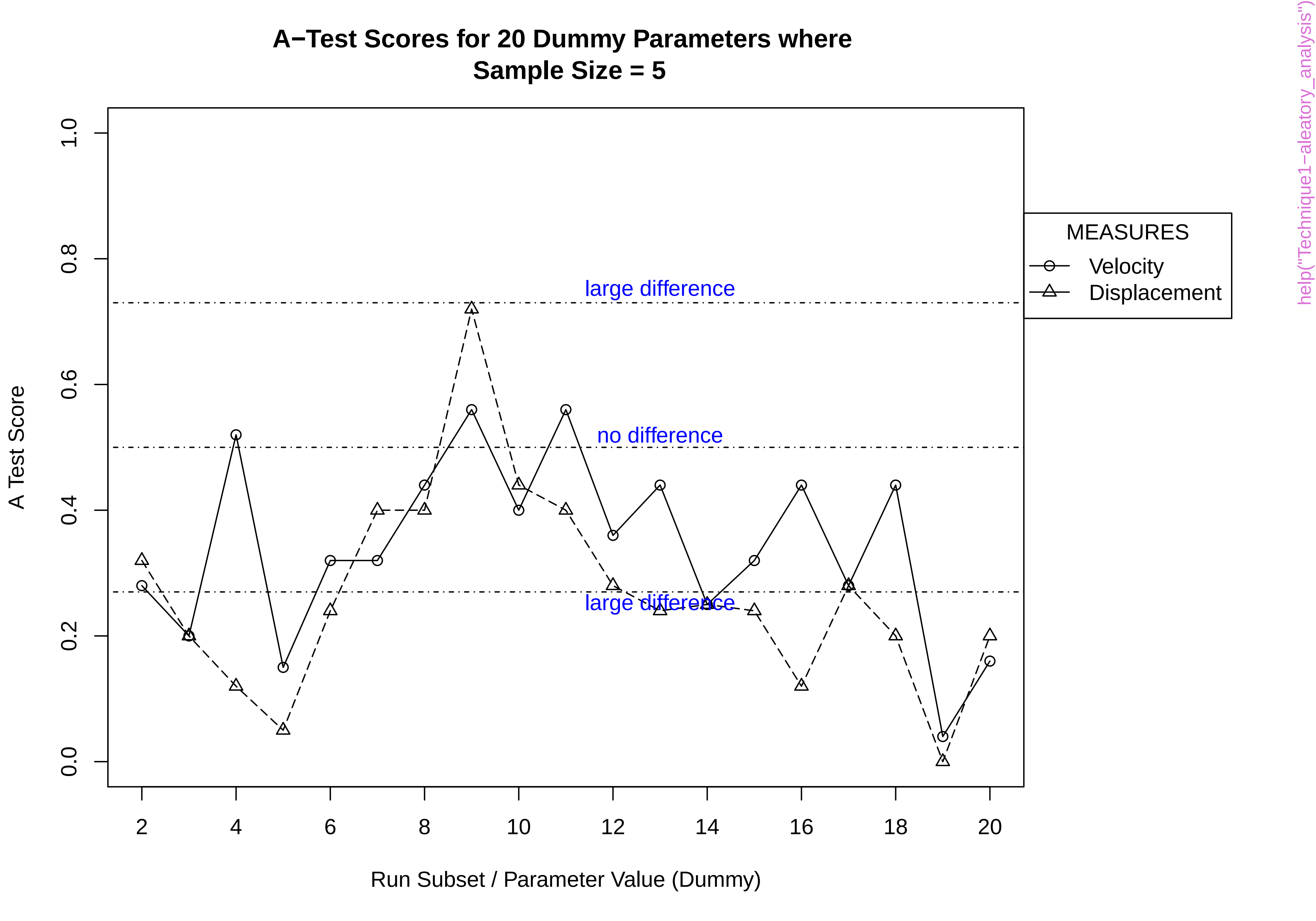
A−Test Scores for 20 Dummy Parameters where
Sample Size = 5
A Test Score
0.0
0.2
0.4
0.6
0.8
1.0
2
4
6
8
10
12
14
16
18
20
Run Subset / Parameter Value (Dummy)
large difference
no difference
large difference
MEASURES
Velocity
Displacement
help("Technique1−aleatory_analysis")
